# Bacterial infection-related glomerulonephritis in patients with diabetes

**DOI:** 10.1111/nep.14222

**Published:** 2023-07-26

**Authors:** Elenjickal Elias John, Sanjeet Roy, Jeethu Joseph Eapen, Reka Karuppusami, Nisha Jose, Selvin Sundar Raj Mani, Joseph Johny, Rizwan Alam, Sabina Yusuf, Athul Thomas, Anna T. Valson, Vinoi George David, Santosh Varughese, Suceena Alexander

**Affiliations:** 1 Department of Nephrology, Christian Medical College, Vellore, India; 2 Department of Pathology, Christian Medical College, Vellore, India; 3 Department of Biostatistics, Christian Medical College, Vellore, India

**Keywords:** diabetic nephropathy, fibrosis, glomerulonephritis, immunopathology, infectious diseases, renal failure

## Abstract

**Aim:**

Diabetic patients are prone to infections, thus making them a unique cohort at risk of developing bacterial infection-related glomerulonephritis (IRGN).

**Methods:**

In total, 1693 adult diabetic patients underwent kidney biopsy between 2005 and 2021 at our tertiary care hospital in South India. Of these, 121 consecutive cases which met criteria of bacterial IRGN were included in this study.

**Results:**

The mean age of the cohort was 53.1 ± 10.1 years and 83/121 (68.5%) were males. Majority (98.3%) had type 2 diabetes for a median duration of 6 (IQR, 2–12) years. The most common sites of infection were skin (47/121, 38.8%) and urinary tract (15/121, 12.4%). Fifty percent (58/121) of patients had underlying advanced diabetic kidney disease (DKD). Isolated C3 deposits (without immunoglobulin) occurred in 66/121 (54.5%) patients predominantly in advanced DKD patients. IgA-dominant glomerulonephritis occurred in only 9/121 (7.4%) patients. Short-course oral steroid was given to 86/121 (71.1%) patients. Steroid related dysglycemia and immunosuppression related infections occurred in 9/61 (14.8%) and 16/61 (26.2%) patients respectively. Of the 90 patients with follow up details >3 months, 46 (51.1%) progressed to kidney failure over a median period of 0.5 (IQR, 0–7.2) months. Patients diagnosed in the latter half of our study period (2013–2021) were older, less commonly presented with fever, had more pronounced hypocomplementemia and severe renal histology predominantly with a ‘starry sky’ immunofluorescence pattern.

**Conclusion:**

Superimposed bacterial IRGN on underlying DKD is associated with poor renal outcomes. Use of short course steroid was associated with significant toxicity.

## 1 Introduction

Bacterial infection-related glomerulonephritis (IRGN) is an immune complex disease which occurs in response to infections.^1^ Diabetic patients are prone to infections making them a unique cohort at risk of IRGN.^2^ Superimposed IRGN on underlying diabetic kidney disease (DKD) is associated with poor renal outcomes.^3^ The role of steroid in diabetic patients with IRGN is controversial.^4^ In spite of diabetes being the most common risk factor for IRGN, there are no previous studies specifically looking into the clinicopathological characteristics of IRGN in diabetic patients. This retrospective study spanning a period of 17 years reports the demographics, clinical features, profile of infections, histopathological characteristics and outcomes in 121 consecutive diabetic patients with kidney biopsy proven bacterial IRGN at a tertiary care centre in South India.

## 2 Materials and Methods

### 2.1 Study design and population

**Figure 1:**
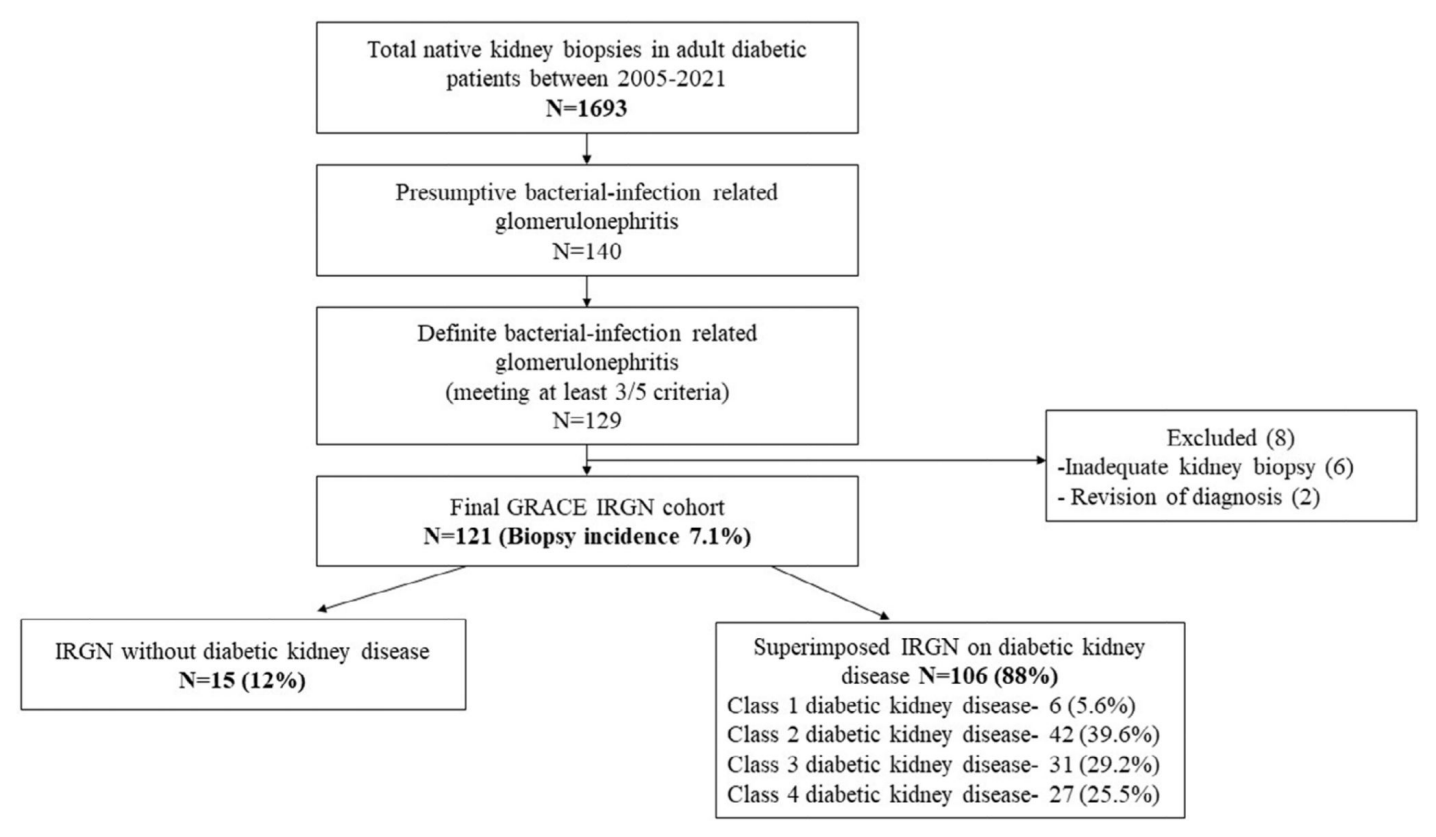
Flowchart of GRACE-IRGN Cohort. DKD, diabetic kidney disease; GRACE-IRGN, glomerular research and clinical experiments-Infection-related glomerulonephritis in diabetics.

This was an observational retrospective biopsy registry cohort [Glomerular Research And Clinical Experiments-Infection Related Glomerulonephritis in diabetics (GRACE-IRGN)] study done in the Department of Nephrology at Christian Medical College, Vellore, the single largest not-for-profit tertiary care centre in South India. In total, 1693 adult diabetic patients underwent native kidney biopsies between January 2005 and December 2021 (Figure 1). Of these, 129 consecutive patients fulfilled at least three of the five criteria for diagnosis of bacterial IRGN.^2^

1. Clinical or laboratory evidence of bacterial infection either prior to or at onset of symptoms of glomerulonephritis (GN).
2. Depressed serum complement levels.
3. Endocapillary proliferative and exudative glomerulonephritis on light microscopy.
4. C3-dominant or codominant glomerular staining on immunofluorescence (IF).
5. Subepithelial ‘humps’ on electron microscopy.

Of the 129 patients, eight were excluded either due to inadequate kidney biopsy tissue (6) or due to revision of diagnosis at follow-up (2). In total, 121 patients were included in this study (biopsy incidence of 7.1%). This study was approved by the institutional review board and a waiver of consent was given by the ethics committee as the data was deidentified and collected retrospectively.

### 2.2 Baseline assessments and treatment

Data on demographic profile, clinical features, biochemical parameters, histopathology variables, treatment details and follow-up outcomes were retrieved from electronic patient records. Proteinuria was assessed from 24-h timed collection, as is the standard practice at our centre. Estimated glomerular filtration rate (eGFR) was calculated by Chronic Kidney Disease Epidemiology Collaboration (CKD-EPI) equation.^5^

Based on timing of IRGN relative to infection, patients were classified into parainfectious, postinfectious and latent infectious GN. Parainfectious GN was defined as presence of concurrent infection at onset of features of GN.^6^ Postinfectious GN was defined as resolution of infection prior to onset of GN.^7^ Latent infectious GN was defined as IRGN occurring with no known infective focus. Latent period (in postinfectious GN) was defined as time (in days) between resolution of infection and onset of features of GN.^7^ Streptococcal infection was identified by isolation of β *hemolytic Streptococcus* in culture or by detection of anti-streptolysin or anti-deoxyribonuclease B antibodies in sera. Skin infections were classified as acute when lesions resolved in 2 weeks (e.g., cellulitis, abscess, pyoderma) or chronic when lesions persisted beyond 2 weeks (e.g., chronic non-healing ulcers). Of the 121 patients in our cohort, 49 were diagnosed between 2005 and 2012 (‘period 1’) and the remaining 72 were diagnosed between 2013 and 2021 (‘period 2’).

All kidney biopsy specimens were processed for light microscopy and immunofluorescence. Serial 3 μm thick tissue sections were stained with haematoxylin and eosin (H&E), periodic acid schiff reagent, masson's trichrome, and jones methenamine silver for light microscopy. Interstitial fibrosis and tubular atrophy (IFTA) was graded semi-quantitatively based on an estimate of percentage of cortex involved into none (0%), mild (1%–25%), moderate (25%–50%) and severe (>50%). DKD was classified as per Renal Pathology Society classification into four classes.^8^ 4-μm cryostat sections were stained with fluorescein-tagged polyclonal rabbit anti-human antisera specific to IgG, IgA, and IgM; complement factors C3 and C1q; and κ and λ light chains (Dako, Carpinteria) for IF studies. Ultrastructural examination (Tecnai T12 Spirit electron microscope) is not routinely done at our centre and was performed only in a minority (6.6%).

The oral steroid protocol at our centre is 2 mg/kg on alternate days for 6–8 weeks followed by a rapid taper over next 6–8 weeks. This was offered to all patients with eGFR <60 mL/min/1.73 m^2^ with no significant chronicity on kidney biopsy and good glycemic status. Intravenous methyl prednisolone pulse (IVMP) was given to all patients with rapidly progressive glomerulonephritis and/or severe renal histology.

### 2.3 Follow-up procedures and study outcomes

Follow-up clinical and outcome data were collected for each review visit until June 2022. Remission of kidney disease was defined as improvement in eGFR by >25% from kidney biopsy and last visit eGFR >90 mL/min/1.73 m^2^ or attainment of baseline eGFR. Stabilisation of kidney disease was defined as change in eGFR not exceeding 25% from kidney biopsy and last visit eGFR >15 mL/min/1.73 m^2^. Worsening of kidney disease was defined as decline in eGFR by >25% from kidney biopsy and last visit eGFR >15 mL/min/1.73 m^2^. Kidney failure was defined as last visit eGFR ≤15 mL/min/1.73 m^2^ or initiation of renal replacement therapy. Complete remission (CR) of proteinuria was defined as proteinuria <0.5 g/day and serum albumin >3.5 g/dL at follow-up. Partial remission (PR) was defined as reduction in proteinuria by at least 50% and to 0.5–3.5 g/day. No remission (NR) was defined as those who failed to attain either CR or PR.

### 2.4 Statistical analyses

Data was presented as mean ± standard deviation (SD) or median [interquartile range (IQR)] or frequency (percentage) according to the types and distribution of variables. Differences among groups of normally distributed variables were analysed by t test or one-way ANOVA. Post hoc comparisons were performed using t test with Bonferroni correction. Differences among groups of non-parametric variables were analysed by Mann–Whitney U test or Kruskal–Wallis test. Categoric variables were compared using Pearson’s chi-squared or Fisher’s exact test. Survival analysis for time to kidney failure was done by Kaplan and Meier using log rank test for comparison between groups. A Cox proportional hazards model was used to identify predictors of kidney failure. Statistical analyses were performed using Statistical Package for Social Sciences software for Windows, version 21.0 (SPSS Inc., Chicago, IL), and graphs were made using Graph Pad Prism 7.0e (Graph Pad Software Inc., San Diego, CA). A *p* value of <.05 was taken as significant.

## 3 Results

### 3.1 Baseline characteristics at kidney biopsy

**Figure 2:**
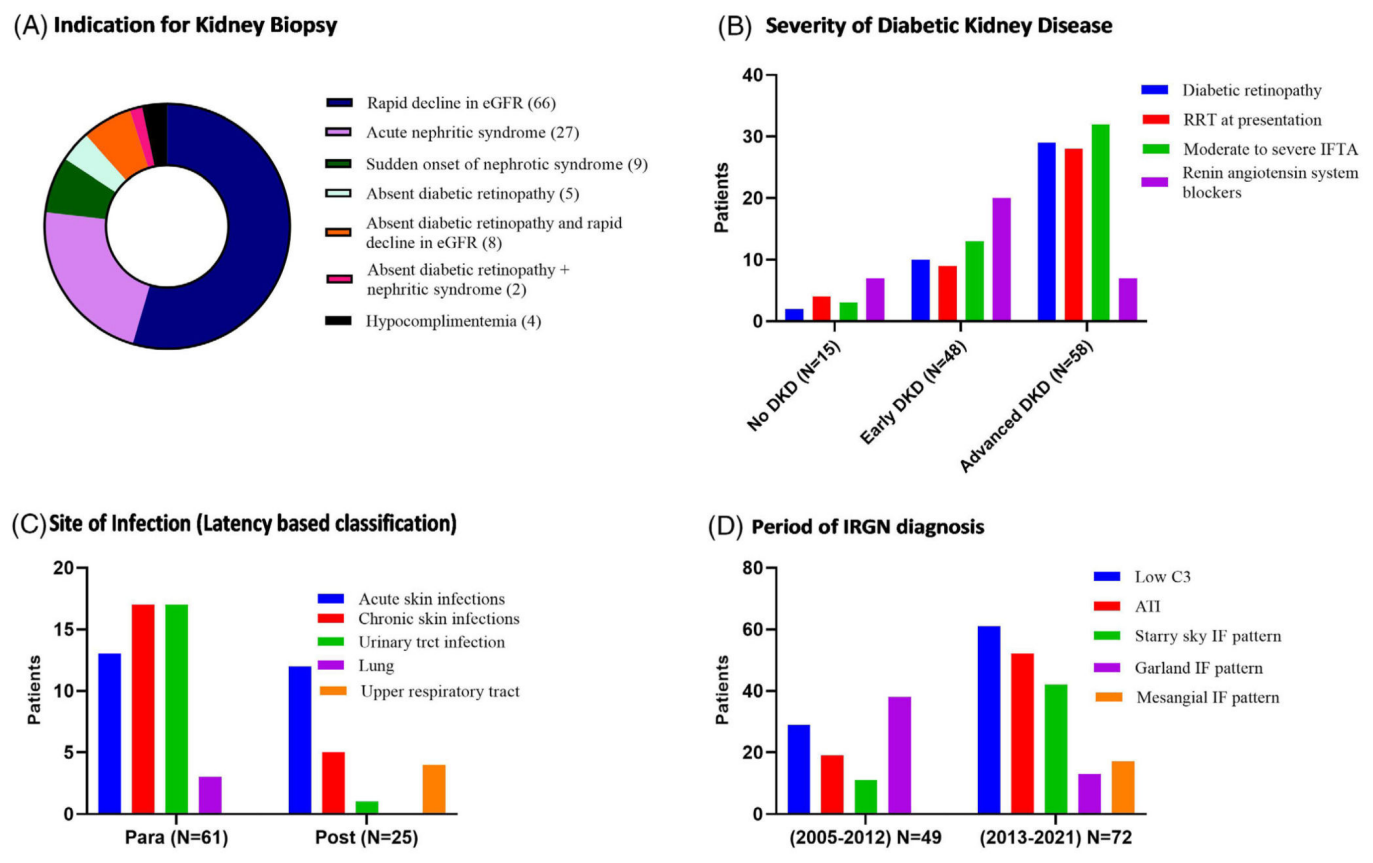
Profile of bacterial infection-related glomerulonephritis in diabetics. (A) Indications for kidney biopsy. (B) Classification based on severity of diabetic kidney disease. (C) Common sites of infection in para and post-infectious glomerulonephritis. (D) Classification based on period of IRGN diagnosis. ATI, acute tubular injury; DKD, diabetic kidney disease; eGFR, estimated glomerular filtration rate; IF, immunofluorescence; IFTA, interstitial fibrosis and tubular atrophy; RRT, renal replacement therapy.

Of the 121 patients, 83 (68.6%) were males and 16 (13.2%) were above 65 years (Table 1). Majority (119/121, 98.3%) had type 2 diabetes with a median duration of 6 (IQR, 2–12) years. Microvascular complications of diabetic retinopathy, peripheral neuropathy and diabetic nephropathy occurred in 41/66 (62.1%), 24/121 (19.8%) and 106/121 (87.6%) patients respectively. The most commonly detected macrovascular complication was coronary artery disease which occurred in 15/121 (12.4%) patients. Almost half of the cohort (64/121) received insulin in addition to oral hypoglycemic agents. The indications for kidney biopsy were rapid decline in eGFR (66/121, 54.5%), nephritic syndrome (27/121, 22.3%), absence of diabetic retinopathy (15/121, 12.4%), sudden onset of nephrotic range proteinuria (9/121, 7.4%) and hypocomplementemia (4/121, 3.3%) (Figure 2A). The common renal manifestations at presentation were hypertension (111/121, 91.7%), edema (102/121, 84.3%) and oliguria (79/121, 65.3%). Fever and congestive heart failure occurred in 32/121 (26.4%) patients each. Hypocomplementemia (low C3) occurred in 90/113 (78.9%) patients. Median eGFR at kidney biopsy was 10.8 (IQR, 6.7–25.4) ml/min/1.73 m^2^ and 41/121 (33.9%) patients required dialysis at biopsy.

### 3.2 Baseline histopathological parameters

**Figure 3:**
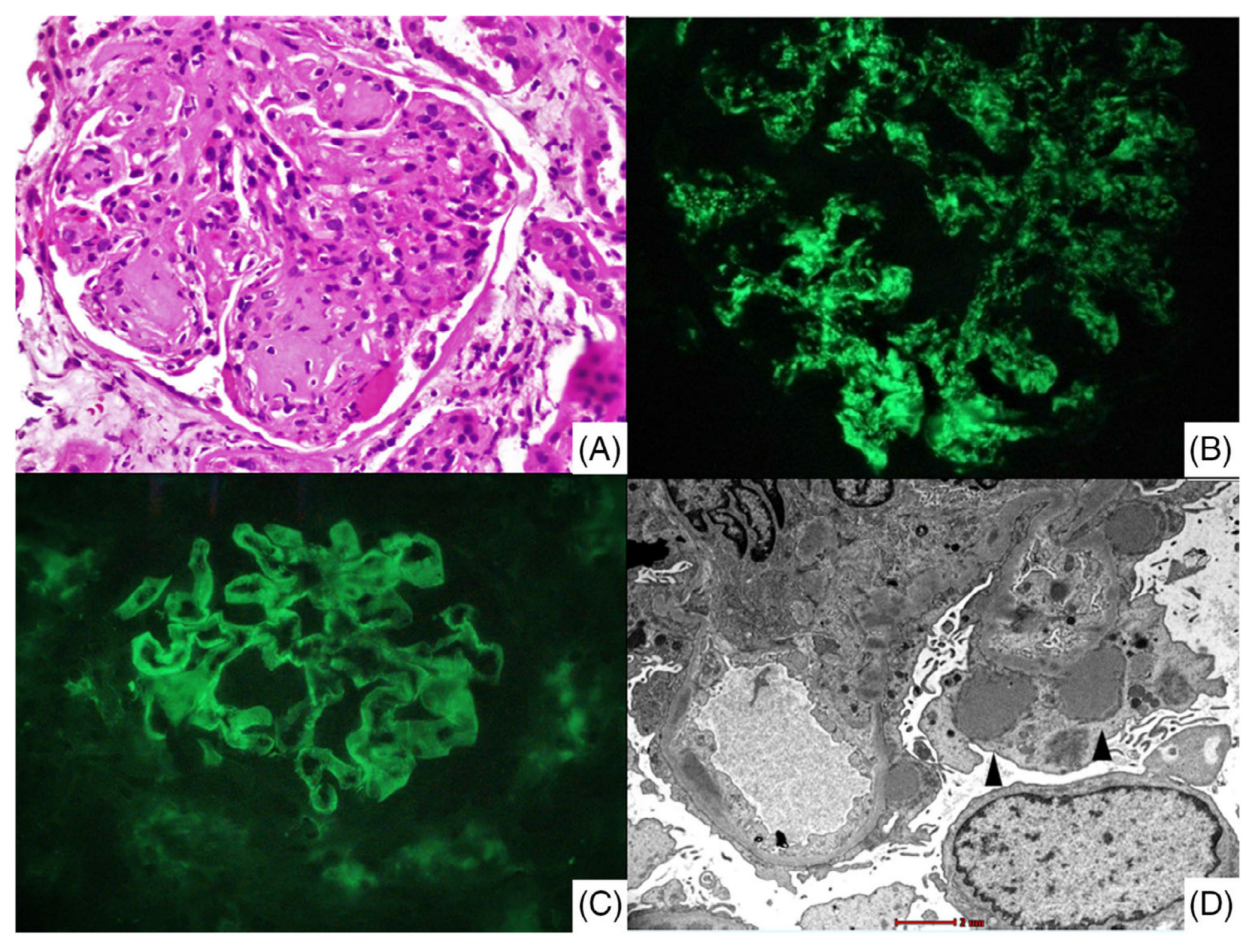
Histopathology of bacterial infection-related glomerulonephritis in diabetics. (A) Glomeruli with changes of diabetic glomerulopathy demonstrated by pronounced mesangial expansion with Kimmelsteil Wilson nodules, along with concomitant global endocapillary hypercellularity and neutrophilic exudation (Haematoxylin and Eosin stain, original magnification ×200). (B) Immunofluorescence microscopy demonstrating granular ‘starry sky’ pattern of C3 along glomerular mesangium and segmental capillary walls (original magnification ×200). (C) Immunofluorescence microscopy demonstrating confluent ‘garland’ pattern of staining of IgG along glomerular capillary walls (original magnification ×200). (D) Glomerulus displaying prominent large discrete subepithelial ‘hump’ like capillary wall electron dense deposits (arrowheads). Few mesangial and subendothelial electron dense deposits are also noted. Overlying podocytes show severe foot process effacement with cytoplasmic microvillous transformation (Transmission EM; original magnification, ×4200).

The median time to kidney biopsy from onset of GN symptoms was 16 (IQR, 10–32) days. The mean ± SD number of glomeruli were 12.1 ± 4.8 (Table 2). The most common light microscopy pattern was diffuse endocapillary proliferative and exudative glomerulonephritis seen in 98/121 (81%) patients (Figure 3A). The commonly detected IF patterns were ‘starry sky’ [53/121 (43.8%)] and ‘garland’ [51/121 (42.1%)] (Figure 3B,C). IgG was the most commonly deposited immunoglobulin in 49/121 (40.5%) patients with a mean staining intensity (MSI) of 0.8 ± 1. C3 deposits were almost universal with a MSI of 2.5 ± 0.7.

Isolated C3 deposits (without immunoglobulin) occurred in 66/121 (54.5%) patients. As compared to patients with both C3 and immunoglobulin deposits, those with isolated C3 deposits had long standing diabetes with more advanced DKD (63.8% vs. 43.8%, *p* = .081) and a lower median (IQR) eGFR [8.9 (6.3–19.9) vs. 18.3 (7.6–33.4) mL/min/1.73 m^2^, *p* = .008] at presentation (Table S1). Patients with both C3 and immunoglobulin deposits had a greater degree of hypocomplementemia (low C3) at presentation [36.1 (IQR, 18.5–72.2) vs. 67.2 (IQR, 33.5–92.7) mg/dL, *p* = .003]. Chronic renal histology like global glomerulosclerosis, IFTA, interstitial inflammation and arterio(lo)sclerosis were significantly more prominent in patients with isolated C3 deposits. Isolated C3 deposits were more commonly detected in ‘period 1’ (69.4% vs. 44.4%, *p* = .009) even though the median (IQR) time to kidney biopsy from onset of GN did not differ between the two time periods [‘period 1’ – 16 (9.5–45) vs. ‘period 2’ – 16.5 (10–31.7) days, *p* = .868]. However, renal outcomes did not differ between the two groups [kidney failure in isolated c3 deposits (29/49, 51.2%) and ‘C3 + immunoglobulin deposits’ (17/41, 41.5%), *p* = .065].

Twenty six percent (32/121) of patients had crescents, and in 4.1% (5/121) this involved >50% of glomeruli. Subepithelial humps on electron microscopy were seen in 7/8 (87.5%) patients (Figure 3D).

### 3.3 Severity of diabetic kidney disease

Majority of patients had underlying DKD [106/121 (87.6%)], of which 58 (54.7%) had advanced DKD [class 3; 31/106 (29.2%) and class 4; 27/106 (25.5%)] (Figure 1). Patients with advanced DKD had longer duration of diabetes, more micro and macrovascular complications, more patients requiring insulin therapy, lower haemoglobin and eGFR at presentation, more chronic histology and isolated C3 deposits on kidney biopsy (Table S2). Patients with advanced DKD were less often treated with either renin-angiotensin-aldosterone system inhibitors (RAASi) or steroids and progressed to kidney failure more frequently (Figure 2B).

### 3.4 Site of infection, infectious agents and latency-based classification

A known source of infection was identified in 86/121 (71.1%) patients, of which 61(50.4%) and 25 (20.7%) patients had parainfectious and postinfectious GN. The most common sites of infection were skin (47/121, 38.8%) and urinary tract (15/121, 12.4%) (Table S3) Patients with acute skin infections had an equal prevalence of parainfectious (13/25, 52%) and postinfectious (12/25, 48%) GN. Whereas, patients with chronic skin infections predominantly had a parainfectious presentation [parainfectious GN; 17/22, 77.3% and postinfectious GN; 5/22 (22.7%), *p* = .016]. Urinary tract (14/15, 93.3%) and lower respiratory tract (3/3, 100%) infections had predilection for parainfectious GN, whereas all cases of upper respiratory tract infections had a postinfectious presentation (Figure 2C). The median latent period in the postinfectious group was 17 (IQR, 12–32.5) days.

An infectious agent was isolated in 52/121 (43%) patients. The most commonly isolated agent was *Streptococcus Pyogenes* (22/121, 18.2%). Gram negative and multiple organisms were isolated in 31/121 (25.6%) and 8/121 (6.6%) patients respectively (Table S3). Staphylococcal and gram-negative infections occurred more commonly in the parainfectious group. Patients with parainfectious GN had more severe presentation and significantly greater number of patients progressed to kidney failure [parainfectious GN; 30/44 (68.2%), postinfectious GN; 6/22 (27.3%) and latent infectious GN; 10/24 (41.7%), *p* = .002] (Table S4).

### 3.5 Drug resistant organisms

Drug resistant organisms were isolated in 24/121 (19.8%) patients. These included gram positive [*methicillin-resistant staphylococcus aureus* (MRSA, 33%), *methicillin-resistant coagulase negative staphylococci* (MRCoNS, 100%), *ampicillin-resistant enterococcus* (ARE, 60%) and *vancomycin-resistant enterococcus* (VRE, 20%)] and gram negative [extended spectrum beta-lactamase (ESBL, 38.7%) and carbapenem-resistant organisms (CRO, 16.1%)] organisms (Table S3). Most of the resistant organisms were isolated from urinary tract (14/24, 58.3%) and skin (11/24, 45.8%). Majority (22/24, 91.7%) had parainfectious presentation.

### 3.6 IgA-dominant IRGN

IgA deposits on immunofluorescence were detected in 17/121 patients, of which 9 biopsies (7.4%) met the diagnostic criteria of IgA dominant IRGN.^2^ Majority were males (8/9, 88.9%) and only one patient was above 65 years. The underlying sites of infection were skin (6/9, 66.7%) and urinary tract (1/9, 11.1%). *Staphylococcus aureus* was isolated in only one case and drug resistant organisms in 3/9 (33.3%) cases. Crescents on biopsy was seen in 4/9 (44.4%) patients. Steroids were used for treatment in 6/9 (66.7%) patients. Forty-percent (2/5) progressed to kidney failure over a median period of 6 (IQR, 3–6) months.

### 3.7 Glycemic status at kidney biopsy

Dysglycemia (HbA1c > 6.5%) at presentation was present in 59/83 (71.1%) patients of which 60% (36/59) received insulin in addition to oral hypoglycemic agents. Dysglycemic patients treated with steroid had more immunosuppression related infections in the follow-up period (35% vs. 9%, *p* = .097). Six patients (10.2%) with dysglycemia died during follow-up (vs. none with normoglycemia).

### 3.8 Treatment

Twenty-one (34/121) percent of patients received RAASi. Oral steroid was given to 86/121 (71.1%) patients, of which 21 (17.4%) received additional IVMP. An add on immunosuppressant was given to 6/121 (4.9%) patients, of which 5 (83.3%) had crescentic IRGN. The most commonly used agent was oral cyclophosphamide (5/6, 83.3%) (Table 3). Patients with resolving patterns of IRGN (mesangial and focal endocapillary proliferation) and chronic changes on biopsy were less commonly treated with steroid. Forty-nine (30/61) and fifty-five (16/29) percent of patients treated with and without steroid progressed to kidney failure (*p* = .602) (Table S5). Steroid related dysglycemia and immunosuppression related infections occurred in 9/61 (14.8%) and 16/61 (26.2%) patients respectively.

### 3.9 Outcomes

Seventy-four percent (90/121) of patients had longitudinal follow-up details of more than 3 months (Table 3). The median duration of follow-up was 6 (IQR, 3–22.5) months. Ninety-two percent (24/26) of patients attained normalization of low C3 over a median period of 2.5 (IQR, 2–4.5) months. Thirty-eight percent (34/90) of patients attained remission of kidney disease over a median period of 2 (IQR, 1–7.5) months. Forty-five percent (24/53) of patients attained complete remission of proteinuria over a median period 6.5 (IQR, 3–13.5) months. Fifty percent (25/50) of patients attained remission of non-visible hematuria over a median period of 10 (IQR, 3–17.5) months. Fifty-one percent (46/90) of patients progressed to kidney failure over a period of 0.5 (IQR, 0–7.2) months. Nine percent (8/90) of deaths occurred during the follow-up period, mostly due to infections (75%).

### 3.10 Predictors of kidney failure

**Figure 4:**
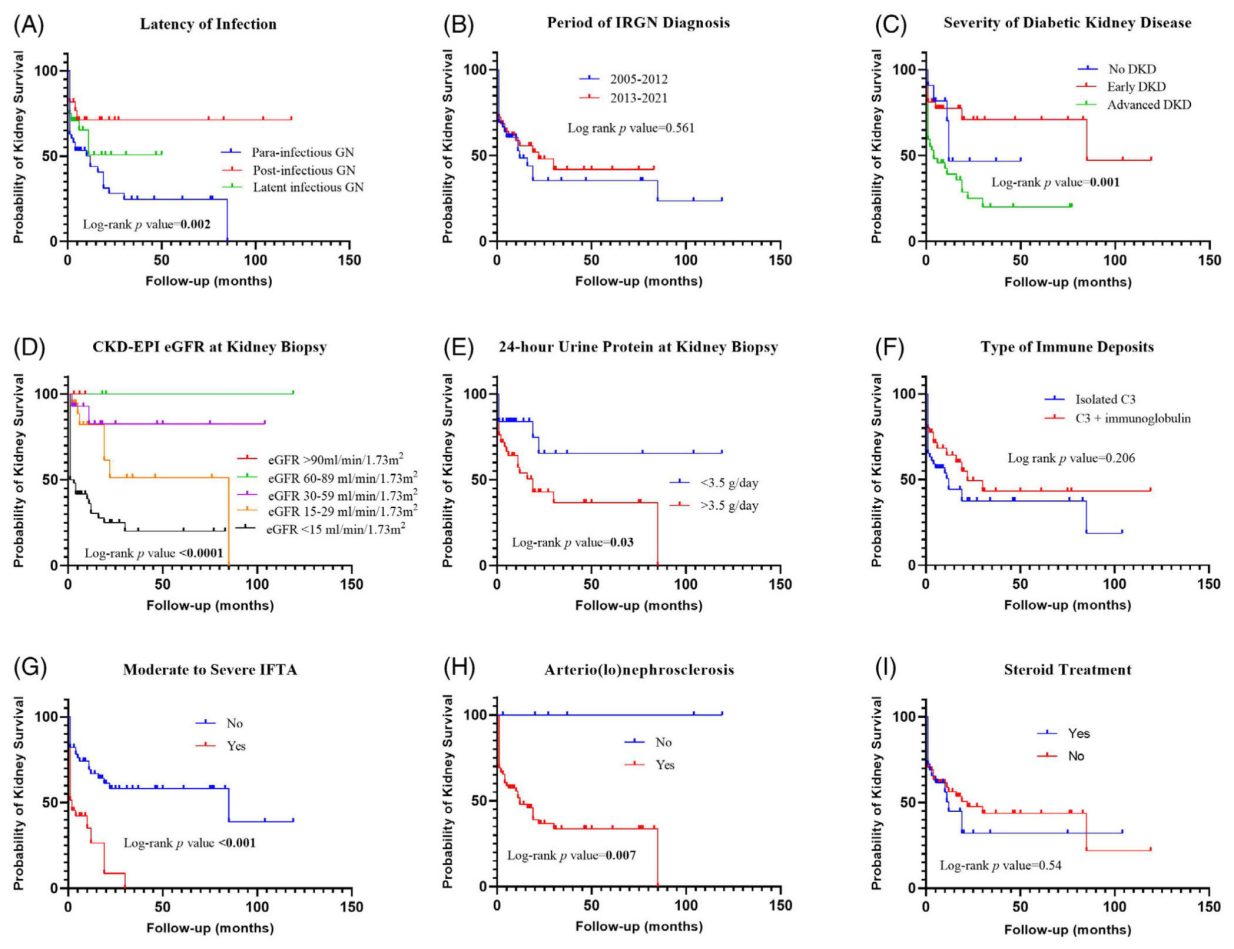
Predictors of kidney failure by Kaplan–Meier survival analysis. CKD-EPI, chronic kidney disease epidemiology collaboration; DKD, diabetic kidney disease; eGFR, estimated glomerular filtration rate; GN, glomerulonephritis; IFTA, interstitial fibrosis and tubular atrophy.

The predictors of kidney failure on univariate analysis were parainfectious GN, eGFR <30 mL/min/1.73 m^2^ at presentation, nephrotic range proteinuria, advanced diabetic kidney disease, moderate to severe IFTA and arterio(lo)nephrosclerosis (Figure 4). A Cox proportional hazard model identified moderate to severe IFTA as the only significant risk factor of kidney failure [HR 2.2 (95% C.I., 1.02–4.8), *p* = .045] (Table S6).

### 3.11 Classification based on time period of IRGN diagnosis

Patients diagnosed in ‘period 2’ were older (55.1 ± 10.3 vs. 50.1 ± 9.2, *p* = .007), less commonly presented with fever (20.8% vs. 34.7%, *p* = .098) and had more pronounced hypocomplementemia (low C3: 88.4% vs. 65.9%, *p* = .005) (Table 4). There were no significant differences noted in sites of infection, infectious agents isolated, drug resistant organisms and latency in the two time periods. Histologically, patients diagnosed in ‘period 2’ had more severe lesions like tuft necrosis, crescents, acute tubular injury and neutrophil rich interstitial infiltrates. The most common IF pattern in ‘period 1’ was ‘garland’ (38/49, 77.6%), whereas ‘starry sky’ (42/72, 58.3%) and ‘mesangial’ patterns (17/72, 23.6%) predominated in ‘period 2’ (*p* = .001). Majority of patients with ‘garland’ (36/51, 70.6%) and ‘mesangial’ patterns (11/17, 64.7%) had isolated C3 deposits. In contrast, majority (34/53, 64.2%) with ‘starry sky’ pattern had C3 along with immunoglobulin deposits (*p* = .001). Patients with ‘garland’ pattern had higher degree of proteinuria at presentation [garland-6 (IQR, 3.3–8.9), starry sky-4.3 (IQR, 1.8–6.5) and mesangial – 4.8 (IQR, 3.3–8.1) g/day, *p* = .045] as well as at last follow-up [NR; garland-11/23 (47.8%), starry sky – 3/22 (13.6%) and mesangial – 2/8 (25%), *p* = .026]. Renal outcomes were however comparable between the three patterns [kidney failure; garland-24/41 (58.5%), starry sky – 18/38 (47.4%) and mesangial-4/11 (36.4%), *p* = .057]. In spite of these differences, baseline proteinuria, eGFR and renal outcomes were comparable between the two periods.

## 4 Discussion

The burden of IRGN in diabetic population is unknown (biopsy incidence is 7.1% in our cohort). To the best of our knowledge, no previous study has addressed this issue in spite of diabetes being the most common predisposing factor, especially in developed countries [10%–44% vs. 3%–21% (developing countries)].^3,9–16^ IRGN remains as one of the most common causes of non-diabetic kidney disease in developing countries [8%–21% (developing countries) vs. 0%–4% (developed countries)]^17–26^ (Table S7). In addition, in most IRGN cohorts underlying diabetes is a risk factor for kidney failure.^3,27,28^

Majority (88%) of our cohort had underling diabetic kidney disease with almost half of them having advanced DKD. Similar findings were noted in previous studies. In a small series of 18 diabetic patients with IRGN, 16 (88.9%) had underlying DKD.^29^ In an elderly cohort of IRGN patients, 43 of 53 (81%) diabetic patients had underlying DKD and 38% had advanced DKD.^30^ In a large biopsy cohort of type 2 diabetic patients, Mazzucco et al. also observed that 70% cases of IRGN occurred in patients with underlying diabetic kidney disease.^31^ Patients with advanced DKD had a higher incidence of micro and macrovascular complications in our study. This predisposes them to develop skin ulcerations with superimposed bacterial infections.

Nasr et al. reported that patients with superimposed IRGN on DKD had histologically more mesangial and subendothelial deposits and less subepithelial deposits. They postulated this to be due to mesangial sclerosis causing inadequate mesangial clearing and glomerular capillary wall thickening impeding formation of large subepithelial humps.^11,32^ This could not be confirmed in our study as electron microscopy was done in only a minority of patients. However, we observed that isolated C3 deposits were more commonly seen in advanced DKD patients. In a biopsy series of 217 diabetic patients where DKD was the only glomerular disease diagnosed, 53.9% had C3 deposits primarily within mesangium. Patients with isolated C3 deposits in this study had lower C3 level, lower serum albumin, more advanced DKD and poor renal outcomes.^33^

Half of our cohort had an ongoing infection at onset of GN. Staphylococcal, gram-negative and drug resistant organisms were more commonly isolated from this parainfectious group. This was similar to findings seen in a pooled analysis of 83 cases of staphylococcal infection related glomerulonephritis wherein all patients had an ongoing infection at onset of GN. Two-thirds of this cohort had MRSA infection.^27^

Kfoury et al. postulated that dysglycemia is associated with more aggressive histological lesions of IRGN based on animal studies.^34^ However, we did not observe any difference in renal histology nor outcomes in patients with and without dysglycemia at presentation. Nasr et al. reported IgA-dominant IRGN as the predominant histopathological pattern in diabetic patients (50%–75%).^32^ Patients with diabetic kidney disease have increased serum IgA levels as well as IgA containing immune complexes. This may be due to more frequent subclinical mucosal infections and impaired IgA clearance. However, IgA dominant IRGN occurred in only 7% of our cohort. This may be explained by differences in infectious agents causing IRGN in our cohort (*Streptococcus pyogenes* and gram-negative infections) as compared to western population (*Staphylococcus aureus*).^11,32^

Patients diagnosed in the latter half of our study period (2013–2021) were older, less commonly presented with fever, had more pronounced hypocomplementemia and severe renal histology predominantly with a ‘starry sky’ IF pattern. ‘Starry sky’ pattern (immunoglobulin and C3) is seen in the early phase of IRGN which over a course of time progresses to ‘mesangial’ pattern pre-dominantly composed of isolated C3 deposits. We observed a decline in ‘garland’ pattern over the years as reported previously.^3,10,11^ However, the sites of infection, infectious agents isolated, drug resistant organism, latency and renal outcomes did not differ between the two time periods. Moroni et al. classified cases of IRGN based on the site of infection into typical infections (pharyngitis and skin infections) and atypical infections (other sites of infection). Over the years, the authors observed a significant increase in atypical infections.^10^ Similarly, a study from Taiwan reported an increase in staphylococcal infections from 50% to 71.4% and decrease in streptococcal infections from 21.4% to 14.3% in IRGN patients diagnosed in time periods 2000–2004 and 2005–2009, respectively.^35^

Fifty-one percent of patients progressed to kidney failure in our cohort. The most significant predictor of kidney failure in our cohort was moderate to severe interstitial fibrosis, which was similar to findings in the French Nationwide Study in IRGN patients with IgA deposits.^13^ Tubulointerstitial fibrosis is mediated by interstitial myofibroblasts which are alpha-smooth muscle actin (α-SMA) positive cells. Oda et al. analysed expression of α-SMA immunohistochemically in kidney biopsy tissue of six patients of post-streptococcal glomerulonephritis at both acute and convalescent phase (3 had complete remission and 3 progressed to CKD). Glomerular α-SMA staining was found to be transient and reversible due to activated mesangial cells, whereas, interstitial α-SMA expression was associated with progression to CKD.^28^ As in other glomerular diseases, the progression of IRGN to kidney failure correlates more with interstitial than glomerular lesions.

Majority of our cohort (71%) received treatment with steroid. However, it did not improve renal outcomes. Twenty-six percent of patients treated with steroid developed infections in the follow-up period which was more seen in patients with dysglycemia at kidney biopsy. In a randomised controlled trial comparing efficacy of corticosteroid versus supportive care in 52 patients of adult IRGN, Arivazhagan et al. observed that immunosuppression related adverse events were significantly more in the treatment arm with no difference in renal outcomes at 6 months. There were seven diabetics included in this study (2 received steroid and 5 supportive care). This study however excluded patients with advanced DKD and a high dose steroid protocol was used, unlike in our study.^4^

In conclusion, bacterial IRGN is an important cause of non-diabetic kidney disease in diabetic patients in developing countries. Over the years, we observe a change in clinical and histopathological presentations of IRGN. Use of steroid is associated with significant toxicity. Renal prognosis is guarded in patients with underlying DKD. The future directions would be incorporation of programs for early diagnosis and aggressive treatment of infections in diabetic patients as an important strategy for primary prevention of chronic kidney disease.

## Supplementary Material

Supplemental table 1-7

## Figures and Tables

**Table 1:** Baseline characteristics of GRACE-IRGN cohort at kidney biopsy.

Baseline characteristics	Entire cohort (*N* = 121)
Sex (*n*, %)	
Men	83 (68.6)
Women	38 (31.4)
Age, years (mean ± SD)	53.1 ± 10.1
Body mass index, kg/m^2^ (mean ± SD)^a^	26.2 ± 4.7
**Diabetes Mellitus**	
Type of Diabetes Mellitus	
Type 1 DM	1 (0.8)
Type 2 DM	119 (98.3)
Gestational DM	1 (0.8)
Duration of diabetes, years [median (IQR)]	6 (2–12)
Microvascular complications (*n*, %)	
Diabetic retinopathy^a^	41 (62.1)
Peripheral Neuropathy	24 (19.8)
Macrovascular complications (*n*, %)	
Coronary artery disease	15 (12.4)
Cerebrovascular accident	1 (0.8)
Peripheral vascular disease	8 (6.6)
HbA1c at biopsy, % (mean ± SD)^a^	7.7 ± 1.8
Antidiabetic treatment (*n*, %)	
Oral hypoglycemic agents	34 (28.1)
Insulin	49 (40.5)
Oral hypoglycemic agents + insulin	15 (12.4)
**Other Comorbidities** (*n*, %)	
Chronic ethanol consumption	6 (5)
Chronic liver disease	4 (3.3)
Malignancies	2 (1.7)
**Blood borne virus infection** (*n*, %)^a^	
HBsAg	3 (2.6)
HCV Ab	0
HIV	1 (0.9)
**Systemic manifestations** (*n*, %)	
Congestive heart failure	32 (26.4)
Hypertensive encephalopathy	0
Fever	32 (26.4)
**Renal manifestations**	
Hypertension (*n*, %)	111 (91.7)
Systolic BP, mmHg (mean ± SD)	143 ± 24
Diastolic BP, mmHg (mean ± SD)	85.5 ± 10.9
Oliguria (*n*, %)	79 (65.3)
Edema (*n*, %)	102 (84.3)
Visible hematuria (*n*, %)	8 (6.6)
**Site of infection** (*n*, %)	
Skin	47 (38.8)
Urinary tract	15 (12.4)
Upper respiratory tract	4 (3.3)
Lung	3 (2.5)
**Causative organisms** (*n*, %)	
*Streptococcus pyogenes*	22 (18.2)
*Staphylococcus aureus*	6 (4.9)
Gram-negative organism	21 (17.3)
Drug resistant organisms	24 (19.8)
**Latency based classification**	
Parainfectious GN	61 (50.4)
Postinfectious GN	25 (20.7)
Latent infectious GN	35 (28.9)
Latent period in postinfectious GN, days [median (IQR)]	17 (12–32.5)
**Serology** (*n*, %)	
Elevated ASO^a^	15 (22.4)
Elevated anti-DNase B^a^	18 (27.7)
**Serum complements**, mg/dL^a^	
Low C3 (*n*, %)	90 (78.9)
Low C4 (*n*, %)	6 (5)
C3 median (IQR)	53.9 (23.8–84.2)
C4 median (IQR)	28.7 (17.3–39.1)
**Urine abnormalities** (*n*, %)^a^	
Nonvisible hematuria	104 (88.9)
Leucocyturia	72 (61.5)
Casts	57 (48.7)
Haemoglobin, g/dL (mean ± SD)	9.8 ± 2
Serum albumin, g/dL (mean ± SD)^a^	2.9 ± 0.7
24-h urine protein, g/day [median (IQR)]	4.7 (2.5–7.7)
**Kidney function at baseline** ^a^	
Serum creatinine, mg/dL [median (IQR)]	1.7 (1.4–2.5)
eGFR CKD-EPI, mL/min/1.73 m^2^ [median (IQR)]	42.3 (23.1–55.9)
**Kidney function at biopsy**	
Serum creatinine, mg/dL [median (IQR)]	5.2 (2.5–7.5)
eGFR CKD-EPI, mL/min/1.73 m^2^ [median (IQR)]	10.8 (6.7–25.4)
eGFR categories, mL/min/1.73 m^2^ (*n*, %)	
≥90	3 (2.5)
60–89	6 (5)
30–59	16 (13.2)
15–29	26 (21.5)
<15	70 (57.9)

Abbreviations: Anti-DNase B, Anti-deoxy-ribonuclease B; ASO, Anti-streptolysin O; BP, Blood pressure; CKD-EPI, Chronic Kidney Disease Epidemiology Collaboration; DM, Diabetes Mellitus; eGFR, Estimated glomerular filtration rate; GN. Glomerulonephritis; GRACE-IRGN, Glomerular Research and Clinical Experiments – Infection Related Glomerulonephritis in diabetics; HbA1c, Glycated haemoglobin; HBsAg, Hepatitis B surface antigen; HCV Ab, Hepatitis C virus antibody; HIV, Human immunodeficiency virus; IQR, Interquartile range; SD, Standard deviation.

a Evaluable patients (*N*) for body mass index = 79, diabetic retinopathy = 66, HbA1c = 83, blood borne virus infection = 115, elevated ASO = 67, anti-DNase B = 65, serum complements = 113, urinary abnormalities = 117, albumin = 115, 24-h urine protein = 99, kidney function at baseline = 71.

**Table 2:** Histopathological parameters of GRACE-IRGN cohort.

Parameters	Entire cohort (*N* = 121)
Time to kidney biopsy from onset of GN, days [median (IQR)]	16 (10–32)
**Light microscopy(*n*** = **121)**	
Number of glomeruli (mean ± SD)	12.1 ± 4.8
Globally sclerosed glomeruli, % [median (IQR)]	22.2 (2.1–43.6)
**Glomerular lesions** (*n*, %)	
Light microscopy pattern	
Mesangial proliferation	4 (3.3)
Focal exudative and endocapillary proliferation	16 (13.2)
Diffuse exudative and endocapillary proliferation	98 (81)
Membranoproliferative pattern	3 (2.5)
Tuft necrosis	5 (4.1)
Crescents	32 (26.4)
>50% crescents	5 (4.1)
Diabetic kidney disease	106 (87.6)
Class 1	6 (5)
Class 2	42 (34.7)
Class 3	31 (25.6)
Class 4	27 (22.3)
**Tubular lesions** (*n*, %)	
Acute tubular injury	71 (58.7)
**Interstitial lesions** (*n*, %)	
Neutrophil infiltration	41 (33.9)
Interstitial inflammation (focal, diffuse)	92. 23 (76, 19)
IFTA moderate to severe	48 (39.7)
**Vascular** (*n*, %)	
Necrosis	1 (0.8)
Arterio(lo)sclerosis	111 (91.7)
**Immunofluorescence staining (*n*** = **121)**	
IF pattern (*n*, %)	
Starry sky	53 (43.8)
Garland	51 (42.1)
Mesangial	17 (14)
Mean staining intensity (*n*, %)	
IgG	0.8 ± 1
IgM	0.1 ± 0.4
IgA	0.2 ± 0.6
C3	2.5 ± 0.7
C1q	0.1 ± 0.3
Isolated C3 staining (*n*, %)	66 (54.5)
IgA dominant GN (*n*, %)	9 (7.4)
**Electron microscopy (*n*** = **8)**	
Subepithelial humps (*n*, %)	7 (87.5)
Subendothelial deposits (*n*, %)	7 (87.5)
Mesangial deposits (*n*, %)	5 (62.5)

Abbreviations: GN, Glomerulonephritis; GRACE-IRGN, Glomerular Research and Clinical Experiments – Infection related glomerulonephritis in diabetics; IF, Immunofluorescence; IFTA, Interstitial fibrosis and tubular atrophy; IQR, Interquartile range; SD, Standard deviation.

**Table 3:** Treatment and outcomes.

Parameters	Entire cohort (*N* = 121)
**Treatment** (*n* = 121) (*n*, %)	
Renin-angiotensin-aldosterone system inhibitors	34 (21.1)
Immunosuppression	86(71.1)
Oral steroid alone	65 (53.7)
Oral steroid plus IVMP	21 (17.4)
Steroids plus add-on immunosuppressant	6 (4.9)
**Outcomes at last follow up (*n*** = **90)**	
>3 months of follow-up (*n*, %)	90 (74.3)
Follow-up duration, months [median (IQR)]	6(3–22.5)
Renal outcomes (*n*, %)	
Remission	34 (37.8)
Stabilization	8 (8.9)
Worsening	2 (2.2)
Kidney failure	46 (51.1)
Proteinuria outcomes (*n*, %)^a^	
Complete remission	24 (45.3)
Partial remission	13 (24.5)
No remission	16 (30.2)
Remission of nonvisible hematuria (*n*, %)^a^	25 (50)
Hypertension outcome (*n*, %)^a^	
Normotension without antihypertensives	15 (24.6)
Normalization of low C3 (*n*, %)^a^	24 (92.3)
Time to kidney events, months [median (IQR)	
Time to normalization of low C3	2.5 (2–4.5)
Time to remission of kidney function	2 (1–7.5)
Time to kidney failure	0.5 (0–7.25)
Time to complete remission of proteinuria	6.5 (3–13.5)
Time to remission of nonvisible hematuria	10 (3–17.5)
Immunosuppression related adverse events (*n*, %)^a^	
Steroid induced dysglycemia	9 (14.8)
Steroid induced cataract	0
Immunosuppression-related infections	16 (26.2)
Death (*n*, %)	8 (8.9)

Abbreviations: IVMP, Intravenous methyl prednisolone; IQR, Interquartile range.

a Evaluable patients (*N*) for proteinuria outcomes = 53, remission of nonvisible hematuria = 50, hypertension outcome = 61, normalization of low C3 = 26; immunosuppression related adverse events = 61.

**Table 4:** Classification based on time period of IRGN diagnosis.

Characteristics	Period 1 (2005–2012) 49/121 (40.5%)	Period 2 (2013–2021) 72/121 (59.5%)	*p* value
Sex (*n*, %)			.327
Men	32 (65.3)	51 (70.8)	
Women	17 (34.7)	21 (29.2)	
Age, years (mean ± SD)	50.1 ± 9.2	55.1 ± 10.3	**.007**
Body mass index, kg/m^2^ (mean ± SD)^a^	25.6 ± 5.2	26.5 ± 4.3	.424
**Hypertension** (*n*, %)	43 (87.8)	68 (94.4)	.313
Systolic BP, mmHg (mean ± SD)	143.1 ± 22.5	143 ± 25.1	.974
Diastolic BP, mmHg (mean ± SD)	87.4 ± 12.3	84.2 ± 9.8	.110
**Diabetes Mellitus**			
Type of Diabetes Mellitus			.501
Type 1 DM	0	1 (1.4)	
Type 2 DM	49 (100)	70 (97.2)	
Gestational DM	0	1 (1.4)	
Duration of diabetes, years [median (IQR)]	6 (3–10)	6 (2–15)	.259
Microvascular complications (*n*, %)			
Diabetic retinopathy^a^	18 (64.3)	23 (60.5)	.802
Peripheral neuropathy	10 (20.4)	14 (19.4)	.896
Diabetic kidney disease			.111
No	8 (16.3)	7 (9.7)	.722
Class 1	2 (4.1)	4 (5.6)	
Class 2	15 (30.6)	27 (37.5)	
Class 3	11 (22.4)	20 (27.8)	
Class 4	13 (26.5)	14 (19.4)	
Macrovascular complications (*n*, %)			
Coronary artery disease	3 (6.1)	12 (16.7)	.084
Cerebrovascular accident	1 (2)	0	.405
Peripheral vascular disease	4 (8.2)	4 (5.6)	.713
HbA1c at biopsy, % (mean ± SD)^a^	7.7 ± 1.9	7.8 ± 1.8	.994
Anti-diabetic treatment (*n*, %)			.893
Oral hypoglycemic agents	13 (26.5)	21 (29.2)	
Insulin	25 (51)	24 (33.3)	
Oral hypoglycemic agents + insulin	4 (8.2)	11 (15.3)	
**Systemic manifestations** (*n*, %)			
Congestive heart failure	14 (28.6)	18 (25)	.680
Fever	17 (34.7)	15 (20.8)	.098
**Site of infection** (*n*, %)			.283
Skin	19 (38.7)	28 (38.9)	
Urinary tract	5 (10.2)	13 (18)	
Upper respiratory tract	1 (2)	3 (4.2)	
**Causative organisms** (*n*, %)			.408
*Streptococcus pyogenes*	6 (12.2)	16 (22.2)	
*Staphylococcus aureus*	4 (8.2)	3 (4.2)	
Gram-negative organisms	5 (10.2)	16 (22.2)	
Drug resistant organisms	9 (18.4)	15 (20.8)	.819
**Latency based classification**			.757
Parainfectious GN	24 (49)	37 (51.4)	
Postinfectious GN	9 (18.4)	16 (22.2)	
Latent infectious GN	16 (32.7)	19 (26.4)	
**Serum complements**, mg/dL^a^			
Low C3 (*n*, %)	29 (65.9)	61 (88.4)	**.005**
Low C4 (*n*, %)	1 (2.3)	5 (7.2)	.403
C3 median (IQR)	73.5 (36.4–95.1)	46.1 (22.4–73.2)	**.007**
C4 median (IQR)	26.4 (15.9–37)	28.5 (19–39.7)	.311
**Urine abnormalities** (*n*, %)^a^			
Non-visible hematuria	44 (89.8)	60 (80.2)	.791
Leucocyturia	25 (51)	47 (69.1)	.056
Casts	34 (69.3)	27 (37.5)	.001
Haemoglobin, g/dL (mean ± SD)	10.1 ± 2.4	9.6 ± 1.8	.277
Serum albumin, g/dL (mean ± SD)^a^	2.9 ± 0.7	2.9 ± 0.6	.765
24-h urine protein, g/day [median (IQR)]^a^	5.5 (2.8–8.1)	4.7 (2.1–7.3)	.371
**Kidney function at biopsy**			
Serum creatinine, mg/dL [median (IQR)]	4.9 (2.6–7.1)	5.5 (2.4–7.9)	.583
eGFR CKD-EPI, mL/min/1.73 m^2^ [median (IQR)]	11.9 (7.4–23.6)	10.3 (6.7–25.9)	.257
eGFR categories, mL/min/1.73 m^2^ (*n*, %)			
≥90	1 (2)	1 (2.3)	.195
60–89	4 (8.2)	0	
30–59	4 (8.2)	7 (15.9)	
15–29	12 (24.5)	7 (15.9)	
<15	28 (57.1)	29 (65.9)	
Time to kidney biopsy from onset of GN, days [median (IQR)]	16 (9.5–45)	16.5 (10–31.7)	.868
**Light microscopy(*n*** = **121)**			
Number of glomeruli (mean ± SD)	12.2 ± 4.7	12.1 ± 4.8	.928
Globally sclerosed glomeruli, % [median (IQR)]	22.2 (0–48.1)	20.4 (1.6–41.5)	.744
**Glomerular lesions** (*n*, %)			
Light microscopy pattern			.151
Mesangial proliferation	0	4 (5.6)	
Focal exudative and endocapillary proliferation	4 (8.2)	12 (16.7)	
Diffuse exudative and endocapillary proliferation	44 (89.8)	54 (75)	
Membranoproliferative pattern	1 (2)	2 (2.8)	
Tuft necrosis	0	5 (6.9)	.080
Crescents	10 (20.4)	22 (30.6)	.294
>50% crescents	1 (2)	4 (5.6)	.647
**Tubular lesions** (*n*, %)			
Acute tubular injury	19 (38.8)	52 (72.2)	**<.001**
**Interstitial lesions** (*n*, %)			
Neutrophil infiltration	12 (24.5)	29 (40.3)	.081
Interstitial inflammation (focal, diffuse)	37, 11 (75.5, 22.4)	55, 12 (76.4, 16.7)	.450
IFTA moderate to severe	20 (40.8)	28 (38.9)	.161
**Vascular** (*n*, %)			
Arteri(lo)nephrosclerosis	44 (89.8)	67 (93.1)	.524
**Immunofluorescence staining (*n*** = **121)**			
IF pattern (*n*, %)			**<.001**
Starry sky	11 (22.4)	42 (58.3)	
Garland	38 (77.6)	13 (18.1)	
Mesangial	0	17 (23.6)	
Isolated C3 staining (*n*, %)	34 (69.4)	32 (44.4)	**.009**
IgA dominant GN (*n*, %)	1 (2)	8 (11.1)	.082
**Treatment and outcomes** (*n* = 121) (*n*, %)			
Renin-angiotensin system inhibitors	14 (28.6)	20 (27.8)	.924
Immunosuppression	37 (75.5)	49 (68.1)	.419
Oral steroid alone	31 (63.3)	34 (47.2)	
Oral steroid plus IVMP	6 (12.2)	15 (20.8)	
Steroids plus add-on immunosuppression	3 (6.1)	3 (4.2)	.685
**Outcomes at last follow up (*n*** = **90)**			
>3 months of follow-up (*n*, %)	36 (73.5)	54 (75)	.850
Follow-up duration, months [median (IQR)]	7.5 (4–32.2)	5.5 (3–22)	.299
Renal outcomes (*n*, %)			.658
Remission	11 (30.6)	23 (42.6)	
Stabilization	3 (8.3)	5 (9.3)	
Worsening	1 (2.8)	1 (1.9)	
Kidney failure	21 (58.3)	25 (46.3)	
Proteinuria outcomes (*n*, %)^a^			**.015**
Complete remission	7 (33.3)	17 (53.1)	
Partial remission	3 (14.3)	10 (31.3)	
No remission	11 (52.4)	5 (15.6)	
Remission of non-visible hematuria (*n*, %)^a^	10 (47.6)	15 (51.7)	.774
Hypertension outcomes (*n*, %)^a^			
Normotension without anti-hypertensives	6 (26.1)	9 (23.7)	.003
Normalization of low C3 (*n*, %)^a^	6 (85.7)	18 (94.7)	.080
Immunosuppression related adverse events (*n*, %)^a^			
Steroid induced dysglycemia	2 (8)	7 (19.4)	.286
Steroid induced cataract	0	0	
Immunosuppression-related infections	5 (20)	11 (30.6)	.393
Death (*n*, %)	2 (5.6)	6 (11.1)	.468

Abbreviations: Anti-DNase B, Anti-deoxy-ribonuclease B; ASO, Anti-streptolysin O, BP, Blood pressure; CKD-EPI, Chronic Kidney Disease Epidemiology Collaboration; DKD, Diabetic kidney disease; DM, Diabetes mellitus; eGFR, Estimated glomerular filtration rate; GN, Glomerulonephritis; HbA1c, Glycated haemoglobin; IF, Immunofluorescence; IFTA, Interstitial fibrosis and tubular atrophy; IQR, Interquartile range; IRGN, Infection related glomerulonephritis; IVMP, Intravenous methyl prednisolone pulse; SD, Standard deviation. Bold denotes *p* < 0.05.

a Evaluable patients (*N*) for Body mass index = 79, diabetic retinopathy = 66, HbA1c = 83, elevated ASO = 67, elevated anti-DNase B = 65, serum complements = 113, urine abnormalities = 117, albumin = 115, 24-h urine protein = 99, proteinuria outcomes = 53, remission of nonvisible hematuria = 50, hypertension outcome = 61, normalization of low C3 = 26; immunosuppression related adverse events = 61.

## Summary at a glance

In this retrospective study spanning a period of 17 years, we report the clinicopathological outcomes in 121 consecutive diabetic patients with biopsy proven bacterial infection-related glomerulonephritis (IRGN). Superimposed bacterial IRGN on underlying diabetic kidney disease is associated with poor renal outcomes. Use of short course steroid was associated with significant toxicity.
