# Supplementary material for: Bacterial infection-related glomerulonephritis in patients with diabetes
Source: Nephrology (Carlton). Author manuscript; Available in PMC 2024 Apr 25. (PMC7615861; doi:10.1111/nep.14222)
Supplement: Supplemental table 1-7 [file EMS195334-supplement-Supplemental_table_1_7.docx]

| **Supplemental table 1: IRGN with ‘isolated C3 deposits’ versus ‘C3 plus immunoglobulin deposits’** | | | |
| --- | --- | --- | --- |
|  | | | |
| **Characteristics** | **Isolated C3 deposits**  66/121 (54.5 %) | **C3 + immunoglobulin deposits**  55/121 (44.5 %) | **p value** |
| Sex (n, %)  Men  Women | 45 (68.2)  21 (31.8) | 38 (69.1)  17 (30.9) | 0.915 |
| Age, years (mean ± SD) | 52.9 ± 9.6 | 53.2 ± 10.8 | 0.875 |
| Body mass index, kg/m² (mean ± SD) ^†^ | 25.3 ± 4.7 | 27.1 ± 4.4 | 0.093 |
| **Hypertension** (n, %)  Systolic BP, mmHg (mean ± SD)  Diastolic BP, mmHg (mean ± SD) | 61 (92.4)  143.3 ± 26.7  85.5 ± 11.8 | 50 (90.9)  143.3 ± 21.5  85.4 ± 10 | 0.763  0.919  0.972 |
| **Diabetes Mellitus**  Type of Diabetes Mellitus  Type 1 DM  Type 2 DM  Gestational DM  Duration of diabetes, years [median (IQR)]  Microvascular complications (n, %)  Diabetic retinopathy ^†^  Peripheral Neuropathy  Diabetic kidney disease  No  Class 1  Class 2  Class 3  Class 4  Macrovascular complications (n, %)  Coronary artery disease  Cerebrovascular accident  Peripheral vascular disease  HbA1c at biopsy, % (mean ± SD) ^†^  Anti-diabetic treatment (n, %)  Oral hypoglycemic agents  Insulin  Oral hypoglycemic agents + insulin | 1 (1.5)  65 (98.5)  0  7 (2.5-12)  26 (68.4)  15 (22.7)  8 (12.1)  3 (5.2)  18 (31)  17 (29.3)  20 (34.5)  8 (12.1)  0  5 (7.6)  7.9 ± 2  16 (24.2)  30 (45.5)  7 (10.6) | 0  54 (98.2)  1 (1.8)  5.5 (2-14.2)  15 (53.6)  9 (16.4)  7 (12.7)  3 (6.3)  24 (50)  14 (29.2)  7 (14.6)  7 (12.7)  1 (1.8)  3 (5.5)  7.5 ± 1.7  18 (32.7)  19 (34.5)  8 (14.5) | 0.705  0.998  0.305  0.493  0.111  0.081  0.920  0.455  0.727  0.296  0.574 |
| **Systemic manifestations** (n, %)  Congestive heart failure  Fever | 21 (31.8)  21 (31.8) | 11 (20)  11 (20) | 0.154  0.154 |
| **Site of infection** (n, %)  Skin  Urinary tract  Upper respiratory tract | 24 (36.4)  8 (12.1)  2 (3) | 23 (41.8)  10 (18.2)  2 (3.6) | 0.959 |
| **Causative organisms** (n, %)  *Streptococcus pyogenes*  *Staphylococcus aureus*  Gram-negative organisms  Drug resistant organisms | 14 (21.2)  4 (6.1)  11 (16.7)  12 (18.2) | 8 (14.5)  3 (5.4)  10 (18.2)  12 (21.8) | 0.970  0.653 |
| **Latency based classification**  Parainfectious GN  Postinfectious GN  Latent infectious GN | 34 (51.5)  12 (18.2)  20 (30.3) | 27 (49.1)  13 (23.6)  15 (27.3) | 0.786 |
| **Period of IRGN diagnosis**  Period 1 (2005-2012)  Period 2 (2013-2021) | 34 (51.5)  32 (48.5) | 15 (27.3)  40 (72.7) | **0.009** |
| **Serum complements**, mg/dl ^†^  Low C3 (n, %)  Low C4 (n, %)  C3 median (IQR)  C4 median (IQR) | 47 (74.6)  4 (6.5)  67.2 (33.5-92.7)  28.2 (17.5-40.6) | 43 (86)  2 (4)  36.1 (18.5-72.2)  27.8 (16.8-35.8) | 0.162  0.690  **0.003**  0.485 |
| **Urine abnormalities** (n, %) ^†^  Non-visible hematuria  Leucocyturia  Casts | 59 (93.7)  35 (55.6)  34 (54) | 45 (83.3)  37 (68.5)  23 (42.6) | 0.138  0.184  0.411 |
| Hemoglobin, g/dL (mean ± SD) | 9.7 ± 2.3 | 9.9 ± 1.7 | 0.669 |
| Serum albumin, g/dL (mean ± SD ^†^ | 2.8 ± 0.6 | 3 ± 0.7 | 0.114 |
| 24-hour urine protein, g/day [median (IQR)] ^†^ | 4.9 (3-8) | 4.4 (1.8-7.4) | 0.309 |
| **Kidney function at biopsy**  Serum creatinine, mg/dL [median (IQR)]  eGFR CKD-EPI, ml/min/1.73 m² [median (IQR)]  eGFR categories, ml/min/1.73 m² (n, %)  ≥90  60-89  30-59  15-29  <15 | 6.2 (3.5-8)  8.9 (6.3-19.9)  0  2 (3)  7 (10.6)  13 (19.7)  44 (66.7) | 3.3 (2.2-6.3)  18.3 (7.6-33.4)  3 (5.5)  4 (7.3)  9 (16.4)  13 (23.6)  26 (47.3) | **0.006**  **0.008**  0.104 |
| Time to kidney biopsy from onset of GN, days [median (IQR)] | 16 (9.7-33.5) | 15 (10-32) | 0.802 |
| **Light microscopy(n=121)**  Number of glomeruli (mean ± SD)  Globally sclerosed glomeruli, % [median (IQR)]  **Glomerular lesions** (n, %)  Light microscopy pattern  Mesangial proliferation  Focal exudative and endocapillary proliferation  Diffuse exudative and endocapillary proliferation  Membranoproliferative pattern  Tuft necrosis  Crescents  >50% crescents  **Tubular lesions** (n, %)  Acute tubular injury  **Interstitial lesions** (n, %)  Neutrophil infiltration  Interstitial inflammation (focal, diffuse)  IFTA moderate to severe  **Vascular** (n, %)  Arteri(lo)nephrosclerosis | 12.8 ± 4.7  26.7 (11.7-50)  0  10 (15.2)  55 (83.3)  1 (1.5)  3 (4.5)  22 (33.3)  2 (3)  35 (53)  19 (28.8)  45, 19 (68.2, 28.8)  36 (54.5)  64 (97) | 11.3 ± 4.8  11.1 (0-35.1)  4 (7.3)  6 (10.9)  43 (78.2)  2 (3.6)  2 (3.6)  10 (18.2)  3 (5.5)  36 (65.5)  22 (40)  47, 4 (85.5, 7.3)  12 (21.8)  47 (85.5) | 0.083  **0.006**  0.110  0.802  0.066  0.658  0.197  0.248  **0.004**  **<0.001**  **0.042** |
| **Immunofluorescence staining (n=121)**  IF pattern (n, %)  Starry sky  Garland  Mesangial  **Electron microscopy** (N=8) (n, %)  Subepithelial humps  Subendothelial deposits  Mesangial deposits | 19 (28.8)  36 (54.5)  11 (16.7)  1 (100)  1 (100)  1 (100) | 34 (61.8)  15 (27.3)  6 (10.9)  6 (85.7)  6 (85.7)  4 (57.1) | **0.001**  0.049 |
| **Treatment and outcomes** (n=121) (n, %)  Renin-angiotensin system inhibitors  Immunosuppression  Oral steroid alone  Oral steroid plus IVMP  Steroids plus add-on immunosuppression | 15 (22.7)  43 (65.2)  32 (48.5)  11 (16.7)  5 (7.6) | 19 (34.5)  43 (78.2)  33 (60)  10 (18.2)  1 (1.8) | 0.161  0.159  0.219 |
|  |  |  |  |
| **Outcomes at last follow up (n=90)**  >3 months of follow-up (n, %)  Follow-up duration, months [median (IQR)]  Renal outcomes (n, %)  Remission  Stabilization  Worsening  Kidney failure  Proteinuria outcomes (n, %) ^†^  Complete remission  Partial remission  No remission  Remission of non-visible hematuria (n, %) ^†^  Hypertension outcomes (n, %) ^†^  Normotension without anti-hypertensives  Normalization of low C3 (n, %) ^†^  Immunosuppression related adverse events  (n, %) ^†^  Steroid induced dysglycemia  Steroid induced cataract  Immunosuppression-related infections  Death (n, %) | 49 (74.2)  5 (3-20.5)  13 (26.5)  5 (10.2)  2 (4.1)  29 (59.2)  8 (34.8)  7 (30.4)  8 (34.8)  10 (47.6)  8 (25.8)  10 (90.9)  2 (6.7)  0  7 (23.3)  4 (8.2) | 41 (74.5)  9 (4-27)  21 (51.2)  3 (7.3)  0  17 (41.5)  16 (53.3)  6 (20)  8 (26.7)  15 (51.7)  7 (23.3)  14 (93.3)  7 (22.6)  0  9 (29)  4 (9.8) | 0.970  0.251  0.065  0.431  0.774  0.454  0.667  0.147  0.772  0.792 |
| Anti-DNase B, Anti-deoxy-ribonuclease B; ASO, Anti-streptolysin O, BP, Blood pressure; CKD-EPI, Chronic Kidney Disease Epidemiology Collaboration; DKD, Diabetic kidney disease; DM, Diabetes mellitus; eGFR, Estimated glomerular filtration rate; GN, Glomerulonephritis; HbA1c, Glycated hemoglobin; IF, Immunofluorescence; IFTA, Interstitial fibrosis and tubular atrophy; IQR, Interquartile range; IVMP, Intravenous methyl prednisolone pulse; SD, Standard deviation.  † Evaluable patients (N) for Body mass index=79, diabetic retinopathy=66, HbA1c=83, elevated ASO=67, elevated anti-DNase B=65, serum complements=113, urine abnormalities=117, albumin=115, 24-hour urine protein=99, kidney function at baseline=71, proteinuria outcomes=53, remission of nonvisible hematuria=50, hypertension outcome=61, normalization of low C3=26; immunosuppression related adverse events=61. | | | |

| **Supplemental table 2: Classification based on severity of diabetic kidney disease** | | | |
| --- | --- | --- | --- |
| **Characteristics** | **Advanced DKD**  **No**  63/121 (52.1%) | **Advanced DKD**  **Yes**  58/121 (47.9%) | **p value** |
| Sex (n, %)  Men  Women | 42 (66.7)  21 (33.3) | 41 (70.7)  17 (29.3) | 0.634 |
| Age, years (mean ± SD) | 53.7 ± 10.7 | 52.4 ± 9.5 | 0.318 |
| Body mass index, kg/m² (mean ± SD) ^†^ | 26.6 ± 5.0 | 25.7 ± 4.3 | 0.374 |
| **Hypertension** (n, %)  Systolic BP, mmHg (mean ± SD)  Diastolic BP, mmHg (mean ± SD) | 58 (92.1)  141.7 ± 20.7  85.1 ± 10.6 | 53 (91.4)  144.5 ± 27.3  85.9 ± 11.3 | 0.891  0.430  0.983 |
| **Diabetes Mellitus**  Type of Diabetes Mellitus  Type 1 DM  Type 2 DM  Gestational DM  Duration of diabetes, years [median (IQR)]  Microvascular complications (n, %)  Diabetic retinopathy ^†^  Mild NPDR  Moderate NPDR  Severe NPDR  PDR  CSME  Peripheral Neuropathy  Diabetic kidney disease  No  Class 1  Class 2  Class 3  Class 4  Macrovascular complications (n, %)  Coronary artery disease  Cerebrovascular accident  Peripheral vascular disease  HbA1c at biopsy, % (mean ± SD) ^†^  Anti-diabetic treatment (n, %)  Oral hypoglycemic agents  Insulin  Oral hypoglycemic agents + insulin | 0  62 (98.4)  1 (1.6)  5 (1.5-10)  12 (36.4)  1 (8.3)  8 (66.7)  1 (8.3)  2 (16.7)  1 (3.1)  9 (14.3)  15 (23.8)  6 (9.5)  42 (66.7)  0  0  3 (4.8)  0  4 (6.3)  7.6 ± 1.9  26 (41.3)  17 (27)  10 (15.9) | 1 (1.7)  57 (98.3)  0  8 (3-14)  29 (87.9)  6 (20.7)  15 (51.7)  2 (6.9)  6 (20.7)  5 (15.2)  15 (25.9)  0  0  0  31 (53.4)  27 (46.6)  12 (20.7)  1 (1.7)  4 (6.9)  7.8 ± 1.8  8 (13.8)  32 (55.2)  5 (8.6) | 0.367  **0.002**  **<0.001**  0.094  0.111  **<.0.001**  **0.008**  0.295  0.518  0.893  **<0.001** |
| **Systemic manifestations** (n, %)  Congestive heart failure  Fever | 16 (25.4)  19 (30.2) | 16 (27.6)  13 (22.4) | 0.785  0.335 |
| **Site of infection** (n, %)  Skin  Urinary tract  Upper respiratory tract | 21 (33.3)  7 (11.1)  3 (4.7) | 26 (44.8)  11 (18.9)  1 (1.7) | 0.281 |
| **Causative organisms** (n, %)  *Streptococcus pyogenes*  *Staphylococcus aureus*  Gram-negative organism  Drug resistant organism | 11 (17.5)  4 (6.3)  10 (15.9)  11 (17.5) | 11 (8.9)  2 (3.4)  11 (18.9)  13 (22.4) | 0.708  0.495 |
| **Latency based classification**  Parainfectious GN  Postinfectious GN  Latent infectious GN | 30 (47.6)  16 (25.4)  17 (27) | 31 (53.4)  9 (15.5)  18 (31) | 0.406 |
| **Serum complements**, mg/dl ^†^  Low C3 (n, %)  Low C4 (n, %)  C3 median (IQR)  C4 median (IQR) | 44 (77.2)  6 (10.7)  53.7 (24.6-84.2)  25.7 (16.2-39.2) | 46 (82.1)  0  54.9 (23.5-85.2)  29.8 (23.1-39.1) | 0.513  **0.027**  0.925  0.088 |
| **Urine abnormalities** (n, %) ^†^  Non-visible hematuria  Leucocyturia  Casts | 56 (91.8)  42 (68.9)  30 (49.2) | 48 (85.7)  30 (53.6)  27 (48.2) | 0.381  0.128  0.575 |
| Hemoglobin, g/dL (mean ± SD) | 10.4 ± 1.7 | 9.1 ± 2.1 | **<0.001** |
| Serum albumin, g/dL (mean ± SD ^†^ | 3 ± 0.6 | 2.8 ± 0.7 | 0.095 |
| 24-hour urine protein, g/day [median (IQR)] ^†^ | 4.5 (1.9-7.8) | 5.3 (2.9-7.6) | 0.342 |
| **Kidney function at biopsy**  Serum creatinine, mg/dL [median (IQR)]  eGFR CKD-EPI, ml/min/1.73 m² [median (IQR)]  eGFR categories, ml/min/1.73 m² (n, %)  ≥90  60-89  30-59  15-29  <15 | 3.9 (1.9-7.1)  16.2 (7.2-37.4)  3 (4.8)  6 (9.5)  11 (17.5)  12 (19)  31 (41.2) | 5.6 (3.3-8.2)  10.3 (6.3-19.1)  0  0  5 (8.6)  14 (24.1)  39 (67.2) | **0.009**  **0.022**  **0.011** |
| Time to kidney biopsy from onset of GN, days [median (IQR)] | 15 (9-30) | 19.5 (11-36) | 0.153 |
| **Light microscopy(n=121)**  Number of glomeruli (mean ± SD)  Globally sclerosed glomeruli, % [median (IQR)]  **Glomerular lesions** (n, %)  Light microscopy pattern  Mesangial proliferation  Focal exudative and endocapillary proliferation  Diffuse exudative and endocapillary proliferation  Membranoproliferative pattern  Tuft necrosis  Crescents  >50% crescents  **Tubular lesions** (n, %)  Acute tubular injury  **Interstitial lesions** (n, %)  Neutrophil infiltration  Interstitial inflammation (focal, diffuse)  IFTA moderate to severe  **Vascular** (n, %)  Arteri(lo)nephrosclerosis | 11.3 ± 4.7  12.7 (0-27.5)  2 (3.2)  9 (14.3)  51 (81)  1 (1.6)  2 (3.2)  20 (31.7)  5 (7.9)  39 (61.9)  22 (34.9)  56, 2 (88.9, 3.2)  16 (25.4)  54 (85.7) | 13 ± 4.7  40 (13.3-54.5)  2 (3.4)  7 (12.1)  47 (81)  2 (3.4)  3 (5.2)  12 (20.7)  0  32 (55.2)  19 (32.8)  36, 21 (62.1, 36.2)  32 (55.2)  57 (98.3) | **0.042**  **<0.001**  0.911  0.670  0.217  0.058  0.467  0.849  **<0.001**  **0.001**  **0.018** |
| **Immunofluorescence staining (n=121)**  IF pattern (n, %)  Starry sky  Garland  Mesangial  Isolated C3 staining (n, %)  IgA dominant GN (n, %) | 31 (49.2)  26 (41.3)  6 (9.5)  29 (46)  7 (11.1) | 22 (37.9)  25 (43.1)  11 (19)  37 (63.8)  2 (3.4) | 0.268  **0.050**  0.109 |
| **Electron microscopy (n=8)**  Subepithelial humps (n, %)  Subendothelial deposits (n, %)  Mesangial deposits (n, %) | 5 (100)  5 (100)  4 (80) | 2 (66.7)  2 (66.7)  1 (33.3) | 0.592 |
| **Treatment and outcomes** (n=121) (n, %)  Renin-angiotensin system inhibitors  Immunosuppression  Oral steroid alone  Oral steroid plus IVMP  Steroids plus add-on immunosuppression | 27 (42.9)  47 (74.6)  29 (46)  18 (28.6)  4 (6.3) | 7 (12.1)  39 (67.2)  36 (62.1)  3 (5.2)  2 (3.4) | **<0.001**  **0.003**  0.549 |
|  |  |  |  |
| **Outcomes at last follow up (n=90)**  >3 months of follow-up (n, %)  Follow-up duration, months [median (IQR)]  Renal outcomes (n, %)  Remission  Stabilization  Worsening  Kidney failure  Proteinuria outcomes (n, %) ^†^  Complete remission  Partial remission  No remission  Remission of nonvisible hematuria (n, %) ^†^  Hypertension outcomes (n, %) ^†^  Normotension without anti-hypertensives  Normalization of low C3 (n, %) ^†^  Immunosuppression related adverse events  (n, %) ^†^  Steroid induced dysglycemia  Steroid induced cataract  Immunosuppression-related infections  Death (n, %) | 43 (68.3)  20 (7-50)  25 (58.1)  4 (9.3)  0  14 (32.6)  22 (66.7)  6 (18.2)  5 (15.2)  13 (43.2)  17 (34.3)  17 (94.4)  5 (17.2)  0  9 (31)  4 (9.3) | 47 (81)  9 (4-22.5)  9 (19.1)  4 (8.5)  2 (4.3)  32 (68.1)  2 (10)  7 (35)  11 (55)  12 (60)  3 (11.5)  7 (87.5)  4 (12.5)  0  7 (21.9)  4 (8.5) | 0.108  0.109  **<0.001**  **<0.001**  0.248  **0.035**  0.080  0.724  0.417  0.895 |
| Anti-DNase B, Anti-deoxy-ribonuclease B; ASO, Anti-streptolysin O, BP, Blood pressure; CKD-EPI, Chronic Kidney Disease Epidemiology Collaboration; CSME, Clinically significant macular edema; DKD, Diabetic kidney disease; DM, Diabetes mellitus; eGFR, Estimated glomerular filtration rate; GN, Glomerulonephritis; HbA1c, Glycated hemoglobin; IF, Immunofluorescence; IFTA, Interstitial fibrosis and tubular atrophy; IQR, Interquartile range; IVMP, Intravenous methyl prednisolone pulse; NPDR, Non-proliferative diabetic retinopathy; PDR, Proliferative diabetic retinopathy; SD, Standard deviation.  † Evaluable patients (N) for body mass index=79, diabetic retinopathy=66, HbA1c=83, elevated ASO=67, elevated anti-DNase B=65, serum complements=113, urinary abnormalities=117, albumin=115, 24-hour urine protein=99, kidney function at baseline=71, proteinuria outcomes=53, remission of nonvisible hematuria=50, hypertension outcome=61, normalization of low C3=26; immunosuppression related adverse events=61. | | | |

| **Supplemental table 3: Site of infection and infectious agents** | | | | | |
| --- | --- | --- | --- | --- | --- |
| **Site of infection** | **No. of patients**  86/121 (71.1%) | **Infectious agent** | **No. of patients**  52/121 (43%) | **Drug resistant organisms** | **No. of patients**  24/121 (19.8**)** |
| Skin  Urinary tract infection  Undifferentiated febrile illness  Upper respiratory tract  Lung  Bone  Gastrointestinal infection  Dental  Eye  Sepsis with unknown focus  Double sites of infection ^†^ | 47 (38.8)  15 (12.4)  12 (9.9)  4 (3.3)  2 (1.6)  2 (1.6)  1 (0.8)  1 (0.8)  1 (0.8)  1 (0.8)  4 (3.3) | **Gram positive:**  *Streptococcus pyogenes* ^‡^  *Staphylococcus aureus*  *Enterococcus*  *CoNS*  **Gram negative:**  *Escherichia coli*  *Klebsiella pneumonia*  *Proteus mirabilis*  *Citrobacter*  *C. diversus*  *C. freundii*  *Pseudomonas aeruginosa*  *Acinetobacter*  *A. baumannii*  *A. junii*  *Salmonella typhi*  *Stenotrophomonas maltophilia*  *Enterobacter*  **Multiple infections:**  Double infections  Triple infections | 22 (18.2)  6 (4.9)  5 (4.1)  3 (3.3)  9 (7.4)  4 (3.3)  3 (2.5)  3 (2.5)  2 (1.6)  1 (0.8)  2 (1.6)  2 (1.6)  1 (0.8)  1 (0.8)  1 (0.8)  1 (0.8)  1 (0.8)  6 (4.9)  2 (1.6) | **Gram positive:**  MRSA  MR-CoNS  ARE  VRE  **Gram negative:**  ESBL  CRO | 2 (33.3)  3 (100)  3 (60)  1 (20)  12 (38.7)  5 (16.1) |
| ARE, Ampicillin resistant enterococcus; CRO, Carbapenem-resistant organisms; CoNS, Coagulase negative staphylococcus; ESBL, Extended spectrum beta-lactamase; MR-CoNS, Methicillin resistant coagulase negative staphylococcus; MRSA, Methicillin resistant staphylococcus aureus; VRE, Vancomycin resistant enterococcus;  † Double sites of infection included skin + urinary tract (3) and skin + lung (1). ‡ Streptococcus pyogenes infection was identified by isolation of β hemolytic streptococcus in culture or by detection of anti-Streptolysin (ASO) or anti-deoxyribonuclease B (anti-DNase B) antibodies in sera. | | | | | |

| **Supplemental table 4: Latency-based classification of bacterial infection related glomerulonephritis in diabetes** | | | | |
| --- | --- | --- | --- | --- |
| **Baseline Characteristics** | **Parainfectious GN**  61/121 (50.4) | **Postinfectious GN**  25/121 (20.7) | **Latent infectious GN**  35/121 (28.9) | **p value** |
| Sex (n, %)  Men  Women | 43 (70.5)  18 (29.5) | 16 (64)  9 (36) | 44 (68.6)  11 (31.4) | 0.841 |
| Age, years (mean ± SD) | 53.7 ± 9.9 | 49.6 ± 10.6 | 54.3 ± 9.8 | 0.161 |
| Body mass index, kg/m² (mean ± SD) ^†^ | 25.3 ± 4.1 | 28.6 ± 6 | 25.9 ± 4.1 | 0.052 |
| **Hypertension** (n, %)  Systolic BP, mmHg (mean ± SD)  Diastolic BP, mmHg (mean ± SD) | 57 (93.4)  142.8 ± 24.5  86 ± 12.6 | 23 (92)  137.6 ± 28.8  85.5 ± 10.1 | 31 (88.6)  147.5 ± 18.4  84.5 ± 8.2 | 0.705  0.296  0.804 |
| **Diabetes Mellitus**  Type of Diabetes Mellitus  Type 1 DM  Type 2 DM  Gestational DM  Duration of diabetes, years [median (IQR)]  Microvascular complications (n, %)  Diabetic retinopathy ^†^  Peripheral Neuropathy  Macrovascular complications (n, %)  Coronary artery disease  Cerebrovascular accident  Peripheral vascular disease  HbA1c at biopsy, % (mean ± SD) ^†^  Anti-diabetic treatment (n, %)  Oral hypoglycemic agents  Insulin  Oral hypoglycemic agents + insulin | 1 (1.6)  60 (98.4)  0  8 (3-15)  22 (78.6)  17 (27.9)  11 (18)  0  5 (8.2)  7.7 ± 1.9  14 (23)  29 (47.5)  8 (32) | 0  24 (96)  1 (4)  4.5 (0.5-10)  6 (40)  3 (12)  3 (12)  0  2 (8)  8.2 ± 1.9  10 (40)  8 (32)  4 (16) | 0  35 (100)  0  6 (2-10)  13 (56.5)  4 (11.4)  1 (2.9)  1 (2.9)  1 (2.9)  7.3 ± 1.7  10 (28.6)  12 (34.3)  5 (14.3) | 0.303  0.107  **0.036**  0.082  0.094  0.290  0.570  0.305  0.546 |
| **Systemic manifestations** (n, %)  Congestive heart failure  Fever | 19 (31.1)  23 (37.7) | 9 (36)  8 (32) | 4 (11.4)  1 (2.9) | 0.052  **0.001** |
| **Site of infection** (n, %)  Skin  Urinary tract  Upper respiratory tract  Lung | 30 (49.2)  17 (27.9)  0  3 (4.9) | 17 (68)  1 (4)  4 (16)  0 | 0  0  0  0 | **<0.001** |
| **Causative organisms** (n, %)  *Streptococcus pyogenes*  *Staphylococcus aureus*  Gram-negative organism  Drug resistant organisms | 12 (19.7)  6 (10)  18 (29.5)  22 (36.1) | 10 (40)  0  3 (12)  2 (8) | 0  0  0  0 | **<0.001** |
| Latent period, days [median (IQR)] | - | 17 (12-32.5) | - |  |
| **Serum complements**, mg/dL ^†^  Low C3 (n, %)  Low C4 (n, %)  C3 median (IQR)  C4 median (IQR) | 42 (72.4)  2 (3.4)  53.8 (22.6-93.2)  28.2 (18.6-39.3) | 17 (73.9)  4 (18.2)  41 (22.4-93.6)  29.5 (14.9-36.1) | 31 (96.9)  0  66.7 (30-80.5)  25.7 (16.5-40.5) | **0.017**  **0.015**  0.502  0.314 |
| **Urine abnormalities** (n, %) ^†^  Nonvisible hematuria  Leucocyturia  Casts | 55 (94.8)  38 (65.5)  26 (44.8) | 21 (87.5)  15 (62.5)  13 (54.2) | 28 (80)  19 (54.3)  18 (51.4) | 0.071  0.560  0.693 |
| Hemoglobin, g/dL (mean ± SD) | 9.5 ± 1.9 | 10 ± 2.6 | 10.2 ± 1.8 | 0.270 |
| Serum albumin, g/dL (mean ± SD) ^†^ | 2.8 ± 0.6 | 3 ± 0.5 | 3 ± 0.8 | 0.309 |
| 24-hour urine protein, g/day [median (IQR)] ^†^ | 5.1 (2.1-7.6) | 3.5 (1.7-6.5) | 4.7 (3.1-8.6) | 0.365 |
| **Kidney function at biopsy**  Serum creatinine, mg/dL [median (IQR)]  eGFR CKD-EPI, ml/min/1.73 m² [median (IQR)]  eGFR categories, ml/min/1.73 m² (n, %)  ≥90  60-89  30-59  15-29  <15 | 5.9 (3.5-8.1)  9.1 (6.2-19.1)  1 (1.6)  1 (1.6)  3 (4.9)  14 (23)  42 (68.9) | 3.3 (1.5-5.7)  18.8 (10.1-43.5)  2 (8)  3 (12)  5 (20)  4 (16)  11 (44) | 3.8 (1.9-7.5)  15.4 (7.2-39.3)  0  2 (5.7)  8 (22.9)  8 (22.9)  17 (48.6) | **0.005***  **0.003***  **0.021** |
| Time to kidney biopsy from onset of GN, days [median (IQR)] | 16 (11-32.5) | 15 (8.5-22) | 20 (11-61) | **0.032***** |
| **Light microscopy(n=121)**  Number of glomeruli (mean ± SD)  Globally sclerosed glomeruli, % [median (IQR)]  **Glomerular lesions** (n, %)  Light microscopy pattern  Mesangial proliferation  Focal exudative and endocapillary proliferation  Diffuse exudative and endocapillary proliferation  Membranoproliferative pattern  Tuft necrosis  Crescents  >50% crescents  Diabetic kidney disease  Class 1  Class 2  Class 3  Class 4  **Tubular lesions** (n, %)  Acute tubular injury  **Interstitial lesions** (n, %)  Neutrophil infiltration  Interstitial inflammation (focal, diffuse)  IFTA moderate to severe  **Vascular** (n, %)  Arterio(lo)nephrosclerosis | 11.4 ± 3.9  25 (10-42.9)  3 (4.9)  7 (11.5)  50 (82)  1 (1.6)  4 (6.6)  18 (29.5)  2 (3.3)  54 (88.5)  3 (5.6)  20 (37)  19 (35.2)  12 (22.2)  35 (57.4)  28 (45.9)  52, 8 (85.2, 13.1)  26 (42.6)  21 (84) | 12.2 ± 5.2  10 (0-34.6)  0  4 (16)  19 (76)  2 (8)  0  6 (24)  0  24 (96)  2 (8.3)  13 (54.2)  4 (16.7)  5 (20.8)  15 (60)  5 (20)  14, 6 (56, 24)  9 (36)  58 (95.1) | 13.3 ± 5.7  30.8 (0-50)  1 (2.9)  5 (14.3)  29 (82.9)  0  1 (2.9)  8 (22.9)  3 (8.6)  28 (80)  1 (3.6)  9 (32.1)  8 (28.6)  10 (35.7)  21 (60)  8 (22.9)  26, 9 (74.3, 25.7)  13 (37.1)  32 (91.4) | 0.157  0.164  0.439  0.446  0.797  0.183  0.453  0.970  **0.018**  **0.001**  0.804  0.237 |
| **Immunofluorescence staining (n=121)**  IF pattern (n, %)  Starry sky  Garland  Mesangial  Isolated C3 staining (n, %)  IgA dominant GN (n, %) | 26 (42.6)  24 (39.3)  11 (18)  34 (55.7)  4 (6.6) | 12 (48)  10 (40)  3 (12)  12 (48)  3 (12) | 15 (42.9)  17 (48.6)  3 (8.6)  20 (57.1)  2 (5.7) | 0.719  0.786  0.729 |
| **Electron microscopy (n=8)**  Subepithelial humps (n, %)  Subendothelial deposits (n, %)  Mesangial deposits (n, %) | 1 (50)  1 (50)  0 | 3 (100)  3 (100)  2 (66.7) | 3 (100)  3 (100)  3 (100) | 0.233 |
| **Treatment and outcomes** (n=121) (n, %)  Renin-angiotensin system inhibitors  Immunosuppression  Oral steroid alone  Oral steroid plus IVMP  Steroids plus add-on immunosuppression | 8 (13.1)  44 (72.1)  30 (49.2)  14 (23)  3 (4.9) | 13 (52)  14 (56)  11 (44)  3 (12)  2 (8) | 13 (37.1)  28 (80)  24 (68.6)  4 (11.4)  1 (2.9) | **<0.001**  0.125  0.664 |
| **Outcomes at last follow up (n=90)**  >3 months of follow-up (n, %)  Follow-up duration, months [median (IQR)]  Renal outcomes (n, %)  Remission  Stabilization  Worsening  Kidney failure  Proteinuria outcomes (n, %) ^†^  Complete remission  Partial remission  No remission  Remission of non-visible hematuria (n, %) ^†^  Hypertension outcome (n, %) ^†^  Normotension without anti-hypertensives  Normalization of low C3 (n, %) ^†^  Immunosuppression related adverse events (n, %) ^†^  Steroid induced dysglycemia  Steroid induced cataract  Immunosuppression-related infections  Death (n, %) | 44 (72.1)  5 (3-32.5)  11 (25)  3 (6.8)  0  30 (68.2)  5 (23.8)  7 (33.3)  9 (42.9)  8 (42.1)  6 (22.2)  5 (100)  6 (20)  0  11 (35.5)  5 (11.4) | 22 (88)  7.5 (3.7-25.5)  15 (68.2)  1 (4.5)  0  6 (27.3)  11 (68.8)  3 (18.8)  2 (12.5)  9 (60)  5 (29.4)  9 (100)  1 (9.1)  0  4 (33.3)  2 (9.1) | 24 (68.6)  7 (4-17.2)  8 (33.3)  4 (16.7)  2 (8.3)  10 (41.7)  8 (50)  3 (18.8)  5 (31.3)  8 (50)  4 (23.5)  10 (83.3)  2 (11.1)  0  1 (5.6)  1 (4.2) | 0.319  0.686  **0.002**  0.090  0.585  0.853  0.006  0.581  0.053  0.608 |
| Anti-DNase B, Anti-deoxy-ribonuclease B; ASO, Anti-streptolysin O; BP, Blood pressure; CKD-EPI, Chronic Kidney Disease Epidemiology Collaboration; DM, Diabetes Mellitus; eGFR, Estimated glomerular filtration rate; GN. Glomerulonephritis; IF, Immunofluorescence; Interstitial fibrosis and tubular atrophy; IQR, Interquartile range; IVMP, Intravenous methylprednisolone pulse; SD, Standard deviation.  One-way ANOVA was used for comparison of means between three groups and the *t* test with Bonferroni correction was used to test significance between groups. *p* value was significant at 0.05 between *one and two, **one and three and ***two and three columns.  † Evaluable patients (N) for body mass index=79, diabetic retinopathy=66, HbA1c=83, blood borne virus infection=115, elevated ASO=67, elevated anti-DNase B=65, serum complements=113, urinary abnormalities=117, albumin=115, 24-hour urine  protein=99, proteinuria outcomes=53, remission of nonvisible hematuria=50, hypertension outcome=61, normalization of low C3=26; immunosuppression related adverse events=61. | | | | |

| **Supplemental table 5: Immunosuppression use in GRACE-IRGN cohort** | | | |
| --- | --- | --- | --- |
| **Parameters** | **Steroid use**  **No**  35/121 (28.9) | **Steroid use**  **Yes**  86/121 (71.1) | **p value** |
| Sex (n, %)  Men  Women | 28 (80)  7 (20) | 55 (64)  31 (36) | 0.085 |
| Age, years (mean ± SD) | 49 ± 9.8 | 54.7 ± 9.8 | 0.005 |
| Body mass index, kg/m² (mean ± SD) ^†^ | 25.4 ± 5.1 | 26.4 ± 4.5 | 0.393 |
| **Hypertension** (n, %)  Systolic BP, mmHg (mean ± SD)  Diastolic BP, mmHg (mean ± SD) | 32 (91.4)  140.6 ± 16.4  84.6 ± 9.8 | 79 (91.9)  144.1 ± 26.6  85.8 ± 11.4 | 0.938  0.387  0.562 |
| **Diabetes Mellitus**  Type of Diabetes Mellitus  Type 1 DM  Type 2 DM  Gestational DM  Duration of diabetes, years [median (IQR)] | 1 (2.9)  33 (94.3)  1 (1)  10 (2.5-14.5) | 0  86 (100)  0  5.5 (2-11.2) | 0.116  0.161 |
| **Systemic manifestations** (n, %)  Congestive heart failure  Fever | 8 (22.9)  6 (17.1) | 24 (27.9)  26 (30.2) | 0.568  0.139 |
| **Site of infection** (n, %)  Skin  Upper respiratory tract  Urinary tract  Lung  Bone  Multiple sites | 19 (54.3)  2 (5.7)  3 (8.6)  3 (8.6)  1 (2.8)  3 (8.6) | 28 (32.5)  2 (2.3)  15 (17.4)  0  1 (1.2)  1 (1.2) | **0.013** |
| **Causative organisms** (n, %)  *Streptococcus pyogenes*  *Staphylococcus aureus*  *CoNS*  *Enterococcus*  Gram-negative organisms  Drug resistant organisms | 10 (28.6)  3 (8.6)  1 (2.9)  1 (2.9)  7 (20)  7 (20) | 12 (13.9)  3 (3.5)  2 (2.3)  4 (4.6)  14 (16.3)  17 (19.8) | 0.276  0.207  0.977 |
| **Latency based classification**  Parainfectious GN  Postinfectious GN  Latent infectious GN | 17 (48.6)  11 (31.4)  7 (20) | 44 (51.2)  14 (16.3)  28 (32.6) | 0.125 |
| **Serum complements**, mg/dL ^†^  Low C3 (n, %)  Low C4 (n, %)  C3 median (IQR)  C4 median (IQR) | 26 (76.5)  4 (11.8)  66.5 (28.2-86.4)  27.5 (17.3-35.4) | 64 (81)  2 (2.6)  53.7 (23.5-84.5)  28.4 (17.1-39.4) | 0.582  0.068  0.454  0.769 |
| **Urine abnormalities** (n, %) ^†^  Non-visible hematuria  Leucocyturia  Casts | 26 (81.3)  18 (56.3)  16 (50) | 78 (91.8)  54 (63.5)  41 (48.2) | 0.183  0.471  0.910 |
| Hemoglobin, g/dL (mean ± SD) | 9.2 ± 1.9 | 10.1 ± 2.0 | **0.036** |
| Serum albumin, g/dL (mean ± SD) ^†^ | 2.9 ± 0.5 | 2.9 ± 0.7 | 0.936 |
| 24-hour urine protein, g/day [median (IQR)] | 4.8 (2.2-7.6) | 4.7 (2.5-7.7) | 0.756 |
| **Kidney function at biopsy**  Serum creatinine, mg/dL [median (IQR)]  eGFR CKD-EPI, ml/min/1.73 m² [median (IQR)] | 4.6 (2.2-7.9)  12.7 (7.2-33.7) | 5.3 (2.7-7.4)  10.8 (6.7-22.4) | 0.771  0.401 |
| Time to kidney biopsy from onset of GN, days [median (IQR)] | 20 (13-33) | 15 (9-32) | 0.374 |
| **Light microscopy(n=121)**  Number of glomeruli (mean ± SD)  Globally sclerosed glomeruli, % [median (IQR)]  **Glomerular lesions** (n, %)  Light microscopy pattern  Mesangial proliferation  Focal exudative and endocapillary proliferation  Diffuse exudative and endocapillary proliferation  Membranoproliferative pattern  Tuft necrosis  Crescents  >50% crescents  **Tubular lesions** (n, %)  Acute tubular injury  **Interstitial lesions** (n, %)  Neutrophil infiltration  Interstitial inflammation (focal, diffuse)  IFTA moderate to severe  **Vascular** (n, %)  Arteri(lo)nephrosclerosis | 12.2 ± 4.2  22.2 (5-46.1)  2 (5.7)  8 (22.9)  25 (71.4)  0 (1)  2 (5.7)  8 (22.9)  0  16 (45.7)  8 (22.9)  22, 9 (62.9, 25.7)  19 (54.3)  32 (91.4) | 12.1 ± 5  23 (0-42.9)  2 (2.3)  8 (9.3)  73 (84.9)  3 (3.5)  3 (3.5)  24 (27.9)  5 (5.9)  55 (64)  33 (38.4)  70, 14 (81.4, 16.3)  29 (33.7)  79 (91.9) | 0.869  0.760  0.093  0.626  0.568  0.320  0.453  0.071  0.102  **0.045**  0.938 |
| **Immunofluorescence staining (n=121)**  IF pattern (n, %)  Starry sky  Garland  Mesangial  Isolated C3 staining (n, %)  IgA dominant IRGN (n, %) | 14 (40)  14 (40)  7 (20)  23 (65.7)  3 (8.6) | 39 (45.3)  37 (43)  10 (11.6)  43 (50)  6 (7) | 0.478  0.115  0.717 |
| **Electron microscopy (n=8)**  Subepithelial humps (n, %)  Subendothelial deposits (n, %)  Mesangial deposits (n, %) | 2 (66.7)  2 (66.7)  2 (66.7) | 5 (100)  5 (100)  3 (60) | 0.390 |
| **Treatment and outcomes** (n=121) (n, %)  Renin-angiotensin-aldosterone system inhibitors  Immunosuppression  Oral steroid alone  Oral steroid plus IVMP  Steroids plus add-on immunosuppression | 9 (25.7)  0  0  0 | 25 (29.1)  65 (71.6)  21 (24.4)  6 (7) | 0.710 |
| **Outcomes at last follow up (n=90)**  >3 months of follow-up (n, %)  Follow-up duration, months [median (IQR)]  Renal outcomes (n, %)  Remission  Stabilization  Worsening  Kidney failure  Proteinuria outcomes (n, %) ^†^  Complete remission  Partial remission  No remission  Remission of non-visible hematuria (n, %) ^†^  Hypertension outcome (n, %) ^†^  Normotension without anti-hypertensives  Normalization of low C3 (n, %) ^†^  Time to kidney events, months [median (IQR)  Time to normalization of low C3  Time to remission of kidney function  Time to kidney failure  Time to complete remission of proteinuria  Time to remission of nonvisible hematuria  Immunosuppression related adverse events (n, %) ^†^  Steroid induced dysglycemia  Steroid induced cataract  Immunosuppression-related infections  Death (n, %) | 29 (82.9)  6 (3.5-18.5)  11 (37.9)  1 (3.4)  1 (3.4)  16 (55.2)  6 (40)  2 (13.3)  7 (46.7)  6 (50)  3 (15.8)  5 (83.3)  2 (2-6)  2 (1-3)  0.5 (0-9.5)  10.5 (1.7-24.2)  4 (1.7-23.2)  0  0  0  1 (3.4) | 61 (70.9)  6 (3-21)  23 (37.7)  7 (11.5)  1 (1.6)  30 (49.2)  18 (47.4)  11 (28.9)  9 (23.7)  19 (50)  12 (28.6)  19 (95)  3 (2-3)  3 (1-12)  0 (0-7.25)  5.5 (3-10.5)  11 (3-18)  9 (14.8)  0  16 (26.2)  7 (11.5) | 0.173  0.761  0.602  0.264  0.629  0.295  0.457  0.661  0.440  0.831  0.546  0.170  0.429 |
| Anti-DNase B, Anti-deoxy-ribonuclease B; ASO, Anti-streptolysin O; BP, Blood pressure; *CoNS, Coagulase negative staphylococcus*; DM, Diabetes Mellitus; GRACE-IRGN, Glomerular Research and Clinical Experiments- Infection Related Glomerulonephritis in diabetics; GN, Glomerulonephritis; IF, Immunofluorescence; IFTA, Interstitial fibrosis and tubular atrophy; IQR, Interquartile range; IVMP, Intravenous methyl prednisolone; SD, Standard deviation.  ^†^ Evaluable patients (N) for body mass index=79, diabetic retinopathy=66, HbA1c=83, elevated ASO=67, elevated anti-DNase B=65, serum complements=113, urinary abnormalities=117, albumin=115, 24-hour urine protein=99, proteinuria outcomes=53, remission of nonvisible hematuria=50, hypertension outcome=61, normalization of low C3=26; immunosuppression related adverse events=61. | | | |

| **Supplemental table 6. Characteristics associated with progression to kidney failure** | | | | | | |
| --- | --- | --- | --- | --- | --- | --- |
| **Risk factors** | **Total number of patients**  **(N)** | **Kidney failure events (n, %)** | **Univariate analysis** | | **Multivariate analysis** | |
|  |  |  | **HR (95% CI)** | **p value** | **HR (95% CI)** | **p value** |
| Age at biopsy |  |  | 1.02 (0.9-1) | 0.142 |  |  |
| Sex  Females  Males | 29  61 | 14 (48.3)  32 (52.5) | 1.37 (0.71-2.61) | 0.344 |  |  |
| Hypertension  No  Yes | 1 (14.3)  45 (54.2) | 7  83 | 4.13 (0.57-30.04) | 0.160 |  |  |
| Type of IRGN  Postinfectious GN  Latent infectious GN  Parainfectious GN | 22  24  44 | 6 (27.3)  10 (41.7)  30 (68.2) | 1.85 (0.67-1.84)  2.89 (1.19-6.99) | **0.046**  0.238  0.018 |  |  |
| Drug resistant organisms  No  Yes | 72  18 | 35 (48.6)  11 (61.1) | 1.376 (0.69-2.72) | 0.359 |  |  |
| HbA1c (%) |  |  | 0.97 (0.71-1.18) | 0.774 |  |  |
| Advanced DKD  No  Yes | 43  47 | 14 (32.6)  32 (68.1) | 2.90 (1.52-5.56) | **0.001** |  |  |
| eGFR at kidney biopsy (ml/min/1.73m²)  ≥30  <30 | 20  70 | 2 (10)  44 (62.9) | 8.74 (2.10-36.41) | **0.003** |  |  |
| 24-hour urine protein (g/day)  <3.5  ≥3.5 | 25  46 | 6 (24)  24 (52.2) | 2.52 (1.02-6.21) | **0.044** |  |  |
| IF pattern  Mesangial  Starry sky  Garland | 11  38  41 | 4 (36.4)  18 (47.4)  24 (58.5) | 1.18 (0.40-3.50)  1.43 (0.49-4.15) | 0.716 |  |  |
| Crescents  No  Yes | 68  22 | 32 (47.1)  14 (63.6) | 1.79 (0.94-3.41) | 0.072 |  |  |
| Global glomerulosclerosis  <50%  ≥50% | 77  13 | 36 (46.8)  10 (76.9) | 2.23 (1.09-4.52) | **0.027** |  |  |
| Moderate to severe IFTA  No  Yes | 57  33 | 21 (36.8)  25 (75.8) | 3.67 (1.97-6.82) | **<0.001** | 2.63 (1.42-4.90) | **0.002** |
| Arterio(lo)nephrosclerosis  No  Yes | 7  83 | 0  46 (55.4) | 30.67 (0.64 to 146.42) | 0.081 |  |  |
| Isolated C3 deposits  No  Yes | 41  49 | 17 (41.5)  29 (59.2) | 1.59 (0.87-2.9) | 0.129 |  |  |
| Treatment with steroid  No  Yes | 29  61 | 16 (55.2)  30 (49.2) | 0.84 (0.46-1.55) | 0.585 |  |  |
| CI Confidence interval; DKD, Diabetic kidney disease; eGFR, Estimated glomerular filtration rate; GN, Glomerulonephritis; LM, Light microscopy; IFTA, Interstitial fibrosis and tubular atrophy; IRGN, Infection related glomerulonephritis. | | | | | | |

| **Supplemental table 7: Burden of Infection related glomerulonephritis in diabetic patients** | | | | | | | | | | |
| --- | --- | --- | --- | --- | --- | --- | --- | --- | --- | --- |
| **Number of IRGN patients in biopsy cohort of diabetic patients** | | | | | | | | | | |
|  | Studies from Indian Subcontinent | | | | | Studies from developed countries | | | | |
| Author | John et al.^17^ | Das et al.^18^ | Soni et al.^19^ | Arora et al.^20^ | Eswarappa et al.^23^ | Bermejo et al.^24^ | Bermejo et al.^21^ | Sharma et al.^22^ | Fontana et al.^25^ | Dias et al.^26^ |
| Country | Vellore, India | Hyderabad, India | Hyderabad, India | Kolkata, India | Bengaluru, India | Spain | Spain | USA | Italy | Portugal |
| Time period | 1985-1993 | 1990-2008 | 2000-2004 | 2011-2012 | 2012-2017 | 1990-2013 | 2002-2014 | 2011 | 2010-2020 | 2016-2018 |
| Total number of diabetic patients | 80 | 75 | 160 | 44 | 236 | 110 | 832 | 620 | 142 | 67 |
| Total number of IRGN patients (%) | 21 | 19 | 17 | 7.7 | 10.4 | 0 | 0 | 1 | 4 | 3 |
| **Number of diabetic patients in biopsy cohort of IRGN patients** | | | | | | | | | |  |
|  | Studies from Indian Subcontinent | | | | | Studies from Western Countries | | | |  |
| Author | John et al.^6^ | Natarajan et al.^14^ | Arivazhagan et al.^4^ | Shankar et al.^15^ | Sanjay et al.^16^ | Montseny et al.^9^ | Moroni et al.^10^ | Nasr et al.^11^ | Nasr et al.^3^ | Standley et al.^13^ |
| Country | Vellore, India | Chennai, India | Chennai, India | Bengaluru, India | Bengaluru, India | France | Switzerland | USA | USA | France |
| Time period | 2005-2017 | 2009-2012 | 2016-2018 | 2018-2021 | 2019 | 1976-1993 | 1979-1999 | 1995-2005 | 2000-2010 | 2007-2017 |
| Total number of IRGN patients | 501 | 102 | 45 | 114 | 73 | 76 | 50 | 86 | 109 | 27 |
| Total number of diabetic patients (%) | 19 | 2.9 | 15.6 | 21.1 | 10 | 33 | 10 | 29 | 49 | 44 |
